# Supplementary material for: Interface preassembly oriented growth strategy towards flexible crystalline covalent organic framework films for OLEDs
Source: Nat Commun. 2025 Apr 8;16:3321. doi: 10.1038/s41467-025-58534-7 (PMC11978764; doi:10.1038/s41467-025-58534-7)
Supplement: Supplementary file 1 — Supplementary Information [file 41467_2025_58534_MOESM1_ESM.pdf]

Supplementary Information

Interface Preassembly Oriented Growth Strategy Towards Flexible Crystalline Covalent Organic Framework Films for OLEDs

Xiang-Chun Li,[a] Hao Sun,[a] Zuqiang Wang,[a] Weijie Yang,[a] Qiaoyu Wang,[a] Chuanrui Wu,[a] Jiajun Chen,[a] Qinchen Jiang,[a] Ling-Jun He,[a] Qian Xue,[a] Wei Huang,[a,b] and Wen-Yong Lai* [a]

[a] State Key Laboratory of Flexible Electronics (LoFE), Institute of Advanced Materials (IAM), School of Chemistry and Life Sciences, Nanjing University of Posts & Telecommunications, 9 Wenyuan Road, Nanjing 210023, China

[b] Frontiers Science Center for Flexible Electronics (FSCFE), MIIT Key Laboratory of Flexible Electronics (KLoFE), Northwestern Polytechnical University, Xi'an 710072, China

*E-mail: iamwylai@njupt.edu.cn

1. Materials and Methods

General Methods. 2,5-dihydroxyterephthalaldehyde (**Ph2OH-2CHO**), 1-bromopentane, methyl chloroacetate, 1,3,5-Tris(4-aminophenyl)benzene (**TAPB-3NH$_2$**), 4,4',4"-(1,3,5-Triazine-2,4,6-triyl) trianiline (**TTr-3NH$_2$**), 4,4'4"-triaminotriphenylamine (**TPA-3NH$_2$**), 4,4',4"-(benzene-1,3,5-triyltris(oxy))trianiline (**TOB-3NH$_2$**), 4,4',4",4'''-(pyrene-1,3,6,8-tetrayl)tetraaniline (**Py-4NH$_2$**), 4,4',4",4'''-(9,9'-spirobi[fluorene]-2,2',7,7'-tetrayl)tetraaniline (**SpF-4NH$_2$**), poly[bis(4-phenyl) (2,4,6-trimethylphenyl)amine (**PTAA**), and all the solvents were purchased from Sigma-Aldrich, J&K or Jilin Extension (China). When necessary, solvents and reagents were purified using standard procedures. All manipulations involving air-sensitive reagents were performed under an atmosphere of dry nitrogen.

Materials Characterization. NMR spectra were recorded on a Bruker Ultra Shield Plus 400 MHz NMR (^{1}H: 400 MHz, ^{13}C: 101 MHz). Mass spectra were measured on Thermo Trace1300-ISQ7000 mass spectrometer. UV-Vis absorption spectra were recorded on a PerkinElmer Lambda 35. X-ray diffraction (XRD) was carried out with a Bruker D8 Advance diffractometer with Cu K_{α} line focused radiation at 40 kV and 40 mA within a 2θ range from 1° to 15° with a step size of 0.02°. Fourier transform infrared (FTIR) spectra were recorded on PerkinElmer Spevtrum Two. The pore structure was assessed from the N_2 isotherm curve measured by a gas adsorption analyzer (Nova Station 4000nova). The Brunauer-Emmett-Teller (BET) method was utilized to calculate the specific surface areas. The COF film was added to the silicon wafer and compacted for measurements. The morphology was investigated by SEM (Hitachi S4800). The surface morphology of the films was investigated by atomic force microscopy (AFM, Bruker Dimension Icon). Thermo-gravimetric analysis (TGA) measurements were done on Shimadzu DTG-60A equipment. KRUSS (DSA20) was used to measure the contact angles.

2. COF-based OLEDs Fabrication and Testing

COF dispersion: The COF film was ground into fine powders, and subsequently, nanosheets are obtained through ultrasonic treatment for 10 min. The solid powders of COF nanocrystalline was obtained after filtration and drying. To prepare the washed glass bottles, a balance is employed to weigh a mass ratio of 9% COF to PTAA. A solution is then prepared by dissolving this mixture in toluene at a concentration of 7 mg/mL. The solution is stirred overnight and placed in an ultrasonic cleaning machine for 30 min to create a uniform dispersion, which is reserved for future use.

Rigid OLEDs: ITO coated glass substrates were cleaned using a soap solution, deionized water, and acetone. The cleaned ITO substrate was treated with O_2 plasma (200 W) for 2 min and the PEDOT:PSS solution was coated with an aqueous 2 μm filter at 2500 rpm for 60 s. Then the substrate was annealed at 100°C for 10 min. Then a COF dispersion containing the mixture of the COF (mass fraction 9%) in PTAA matrix (7 mg/mL concentration) was spin-coated for 30 s at 1500 rpm. Then it was further annealed at 95°C for 10 min. Then it was placed into the vacuum evaporation chamber for evaporation. The TPBi layer (45 nm) and LiF layer (1 nm) was evaporated using vacuum evaporation chamber. Finally, the Al electrode was evaporated. The structure of the OLEDs is ITO/PEDOT:PSS (50 nm)/PTAA:COF (100 nm)/TPBi (45 nm)/LiF (1 nm)/Al (100 nm).

Flexible OLEDs: ITO coated flexible polyethylene terephthalate (PET) substrates were cleaned using ethyl alcohol. The cleaned flexible ITO substrate was dried with N_2 and the PEDOT:PSS solution was coated with an aqueous 2 μm filter at 2500 rpm for 60 s. Then a solution containing the mixture of the COF (mass fraction 9%) in PTAA matrix (7 mg/mL concentration) was spin-coated for 30 s at 1500 rpm. Then it was placed into the vacuum evaporation chamber for evaporation. The TPBi layer (45 nm) and LiF layer (1 nm) was evaporated using vacuum evaporation chamber.

The luminance-current-voltage characteristics of the devices were recorded using a combination of a

Keithley source-meter (model 2602) and a calibrated luminance meter. A spectrometer (PR655) was used to measure the EL spectra and Commission International de l'Eclairage (CIE) coordinates of the devices. All the devices were characterized without encapsulation, and all the measurements were carried out under ambient conditions at room temperature. The thickness of the organic films was measured by using a spectroscopic ellipsometry (α-SE, J.A. Wollam Co. Inc.). All the measurements were carried out at room temperature under ambient conditions.

3. Synthetic Procedures

Synthesis of **Ph2Pe-2CHO**: Ph2OH-2CHO (664.5 mg, 4 mmol) and anhydrous K_2CO_3 (1105.6 mg, 8 mmol) were accurately weighed and transferred into a two-mouth flask. The flask was then sealed, and nitrogen was purged to remove moisture, followed by heating to ensure dryness. This nitrogen purge was repeated three times before adding 1-bromopentane (1449.9 mg, 9.6 mmol). Anhydrous acetonitrile (30 mL), which had been bubbled with nitrogen, was subsequently added to the flask. The reaction mixture was then heated in an oil bath at 80°C for 24 hours. After the reaction, the mixture was extracted three times. The organic layer was then concentrated under vacuum, and the residue was washed with the filter three times. The crude product was then separated and purified using a 200-300 mesh silica gel column with an elution solvent of ethyl acetate and petroleum ether in a ratio of 1:4. Finally, the product **Ph2Pe-2CHO** was obtained after vacuum drying, yielding 860.2 mg (70%). ^{1}H NMR (400 MHz, $CDCl_3$, ppm) δ 10.55 (s, 2H), 7.45 (s, 2H), 4.11 (t, J = 6.5 Hz, 4H), 1.90-1.84 (m, 4H), 1.50-1.40 (m, 8H), 0.96 (t, J = 7.1 Hz, 6H). ^{13}C NMR (101 MHz, $CDCl_3$, ppm) δ 189.53, 155.31, 129.36, 111.70, 69.32, 28.82, 28.24, 22.46, 14.06. GC-MS (*m*/*z*): Calcd for $C_{18}H_{26}O_4$, Exact Mass: 306.18, Mol. Wt.: 306.40, Found: 306.3.

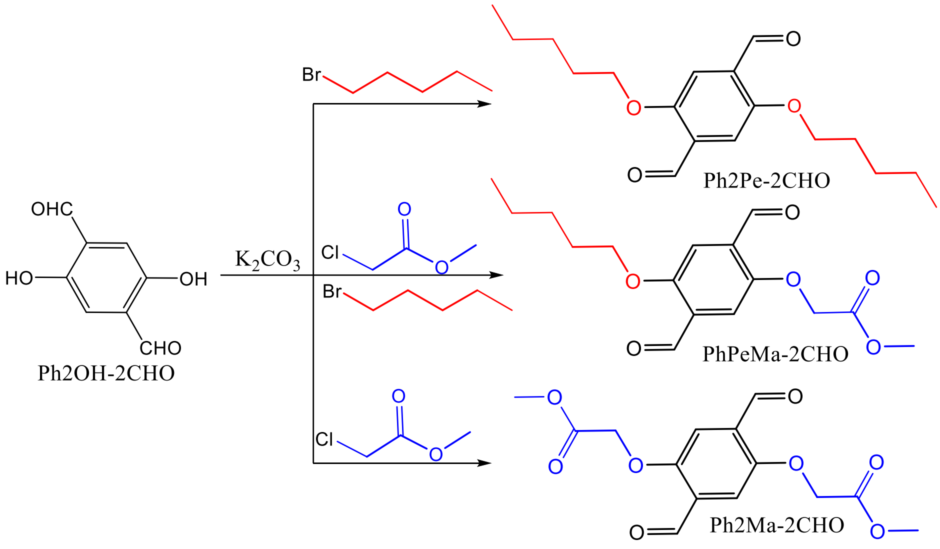

Figure S1. The synthetic routes of **Ph2Pe-2CHO**, **PhPeMa-2CHO** and **Ph2Ma-2CHO**.

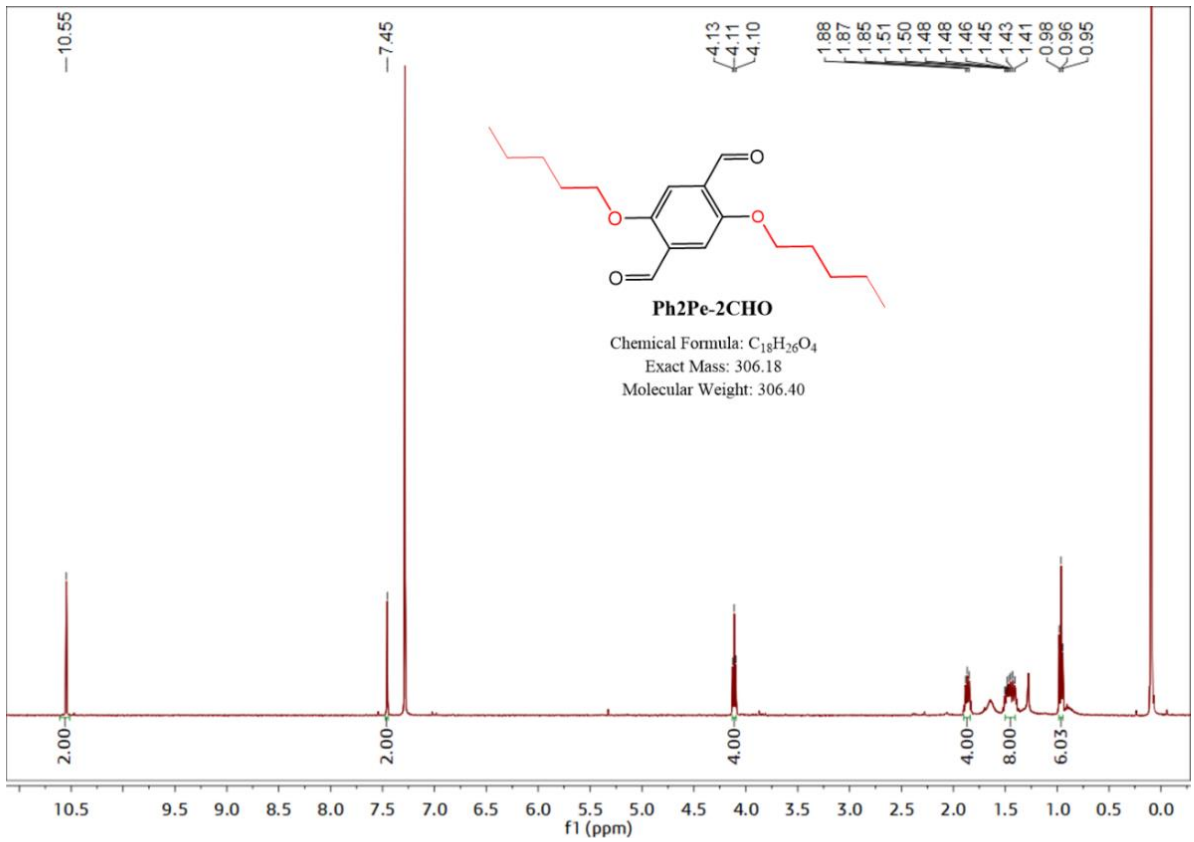

Figure S2. ^{1}H NMR of **Ph2Pe-2CHO**.

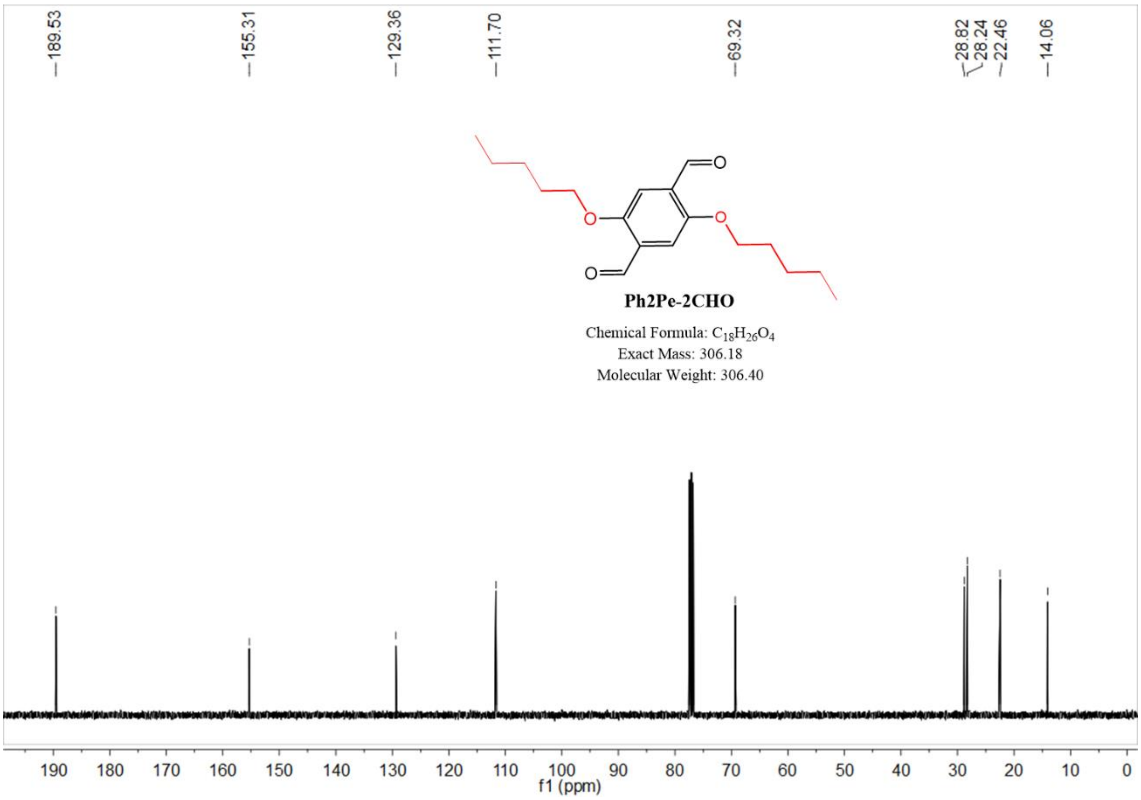

Figure S3. ^{13}C NMR of **Ph2Pe-2CHO**.

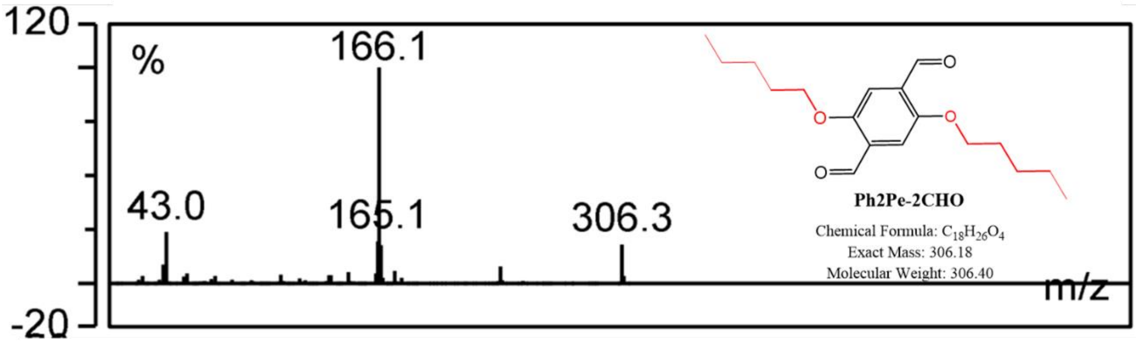

Figure S4. Mass spectrum of **Ph2Pe-2CHO**.

Synthesis of **PhPeMa-2CHO**: Ph2OH-2CHO (664.5 mg, 4 mmol) and anhydrous K_2CO_3 (1105.6 mg, 8 mmol) were accurately weighed and transferred into a two-mouth flask. The flask was then sealed, and nitrogen was purged to remove moisture, followed by heating to ensure dryness. This nitrogen purge was repeated three times before adding 1-bromopentane (724.9 mg, 4.8 mmol) and methyl chloroacetate (651.1 mg, 6 mmol). Anhydrous acetonitrile (30 mL), which had been bubbled with nitrogen, was subsequently added to the flask. The reaction mixture was then heated in an oil bath at 80°C for 24 hours. After the reaction, the mixture was extracted three times. The organic layer was

then concentrated under vacuum, and the residue was washed with the filter three times. The crude product was then separated and purified using a 200-300 mesh silica gel column with an elution solvent of ethyl acetate and petroleum ether in a ratio of 1:3. Finally, the product **PhPeMa-2CHO** was obtained after vacuum drying, yielding 381.8 mg (31%). ^{1}H NMR (400 MHz, $CDCl_3$, ppm) *δ* 10.52 (s, 1H), 10.43 (s, 1H), 7.41 (s, 1H), 7.27 (s, 1H), 4.72 (s, 2H), 4.04 (t, J = 6.5 Hz, 2H), 3.74 (s, 3H), 1.80-1.76 (m, 2H), 1.35 (dd, J = 11.4, 3.4 Hz, 4H), 0.88 (d, J = 7.1 Hz, 3H). ^{13}C NMR (101 MHz, $CDCl_3$, ppm) *δ* 189.18, 189.10, 168.58, 156.24, 153.75, 130.17, 129.06, 112.09, 112.07, 69.39, 66.02, 52.49, 28.77, 28.22, 22.44, 14.04. GC-MS (*m*/*z*): Calcd for $C_{16}H_{20}O_6$, Exact Mass: 308.13, Mol. Wt.: 308.33, Found: 308.2.

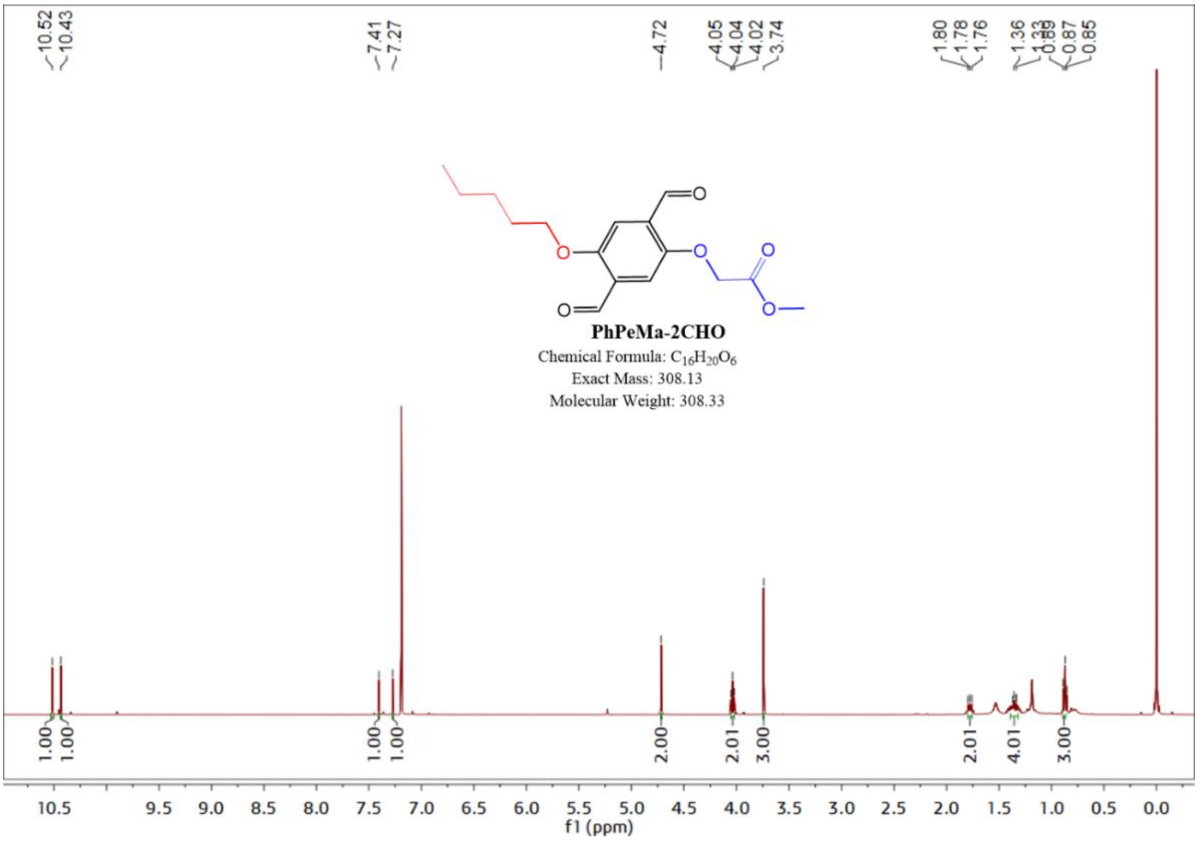

Figure S5. ^{1}H NMR of **PhPeMa-2CHO**.

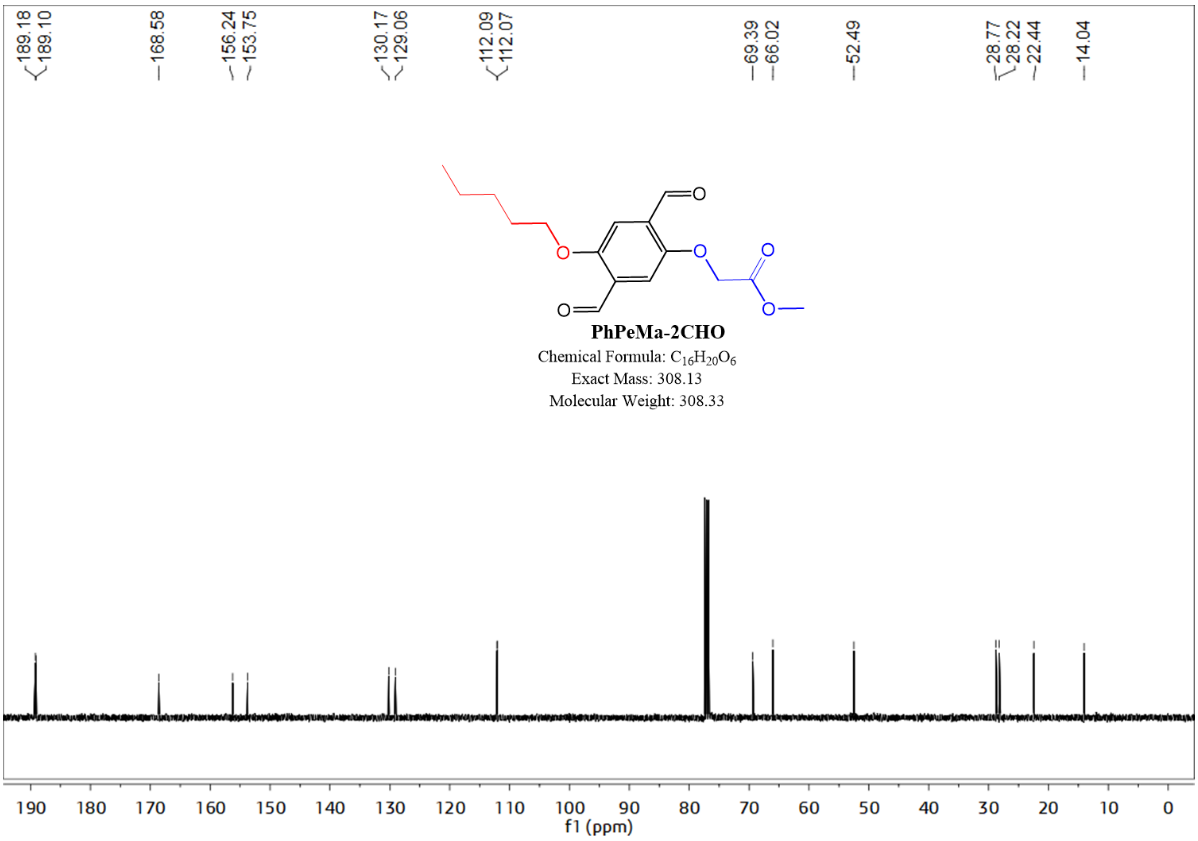

Figure S6. ^{13}C NMR of **PhPeMa-2CHO**.

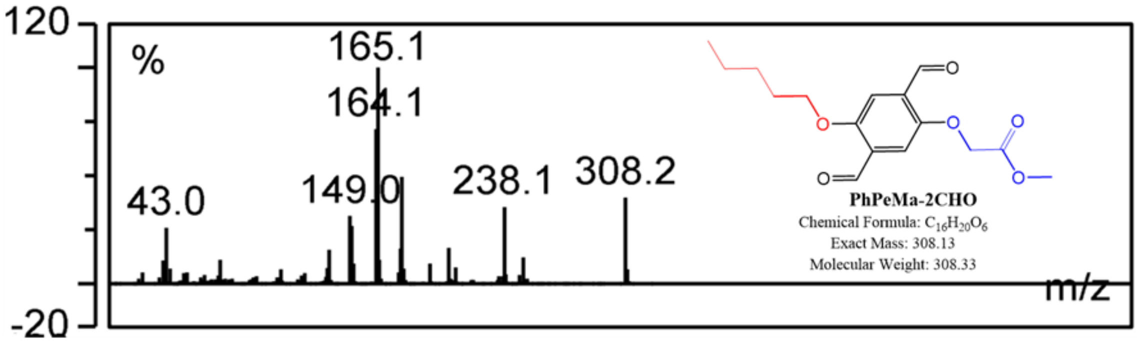

Figure S7. Mass spectrum of **PhPeMa-2CHO**.

Synthesis of **Ph2Ma-2CHO**: Ph2OH-2CHO (664.5 mg, 4 mmol), anhydrous K_2CO_3 (1105.6 mg, 8 mmol) were accurately weighed and transferred into a two-mouth flask. The flask was then sealed, and nitrogen was purged to remove moisture, followed by heating to ensure dryness. This nitrogen purge was repeated three times before adding methyl chloroacetate (1041.6 mg, 9.6 mmol). Anhydrous acetonitrile (30 mL), which had been bubbled with nitrogen, was subsequently added to the flask. The reaction mixture was then heated in an oil bath at 80°C for 24 hours. After the reaction,

the mixture was extracted three times. The organic layer was then concentrated under vacuum, and the residue was washed with the filter three times. The crude product was then separated and purified using a 200-300 mesh silica gel column with an elution solvent of ethyl acetate and petroleum ether in a ratio of 1:1. Finally, the product **Ph2Ma-2CHO** was obtained after vacuum drying, yielding 844.1 mg (68%). ^{1}H NMR (400 MHz, $CDCl_3$, ppm) δ 10.59 (s, 2H), 7.40 (s, 2H), 4.82 (s, 4H), 3.84 (s, 6H). ^{13}C NMR (101 MHz, $CDCl_3$, ppm) δ 188.74, 168.43, 154.68, 129.87, 112.44, 65.92, 52.41. GC-MS (*m*/*z*): Calcd for $C_{14}H_{14}O_8$, Exact Mass: 310.07, Mol. Wt.: 310.26, Found: 310.2.

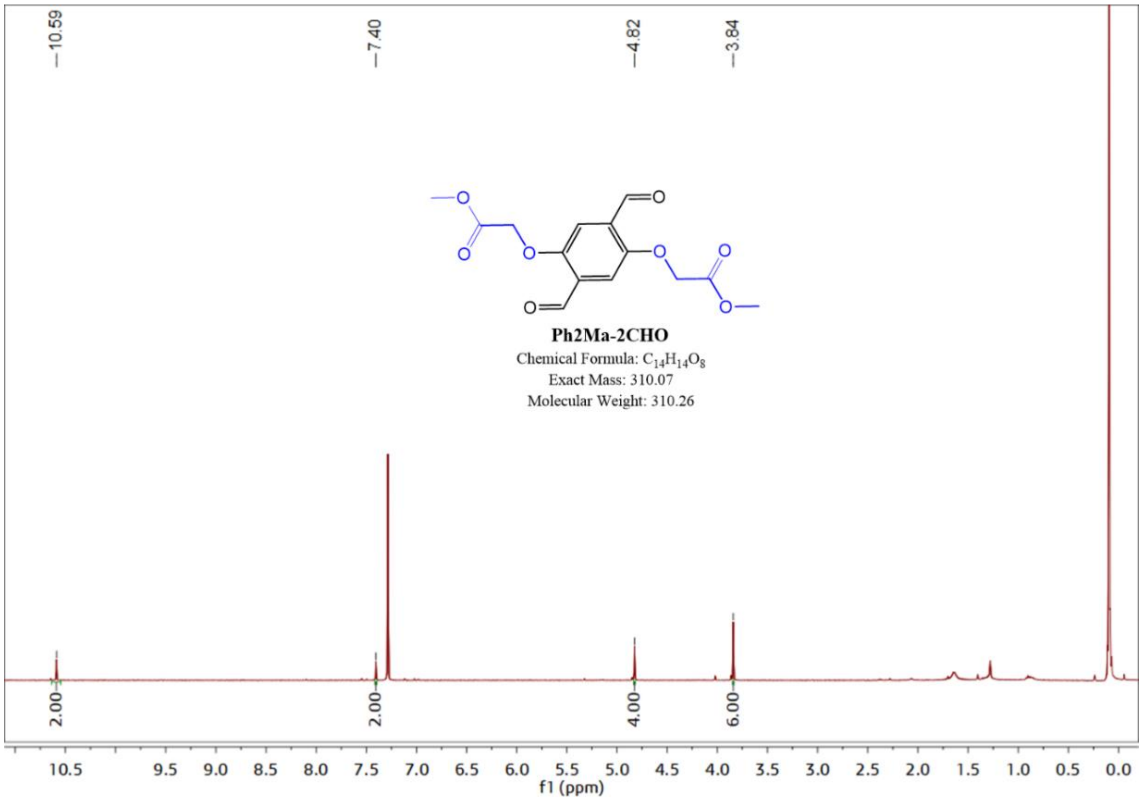

Figure S8. ^{1}H NMR of **Ph2Ma-2CHO**.

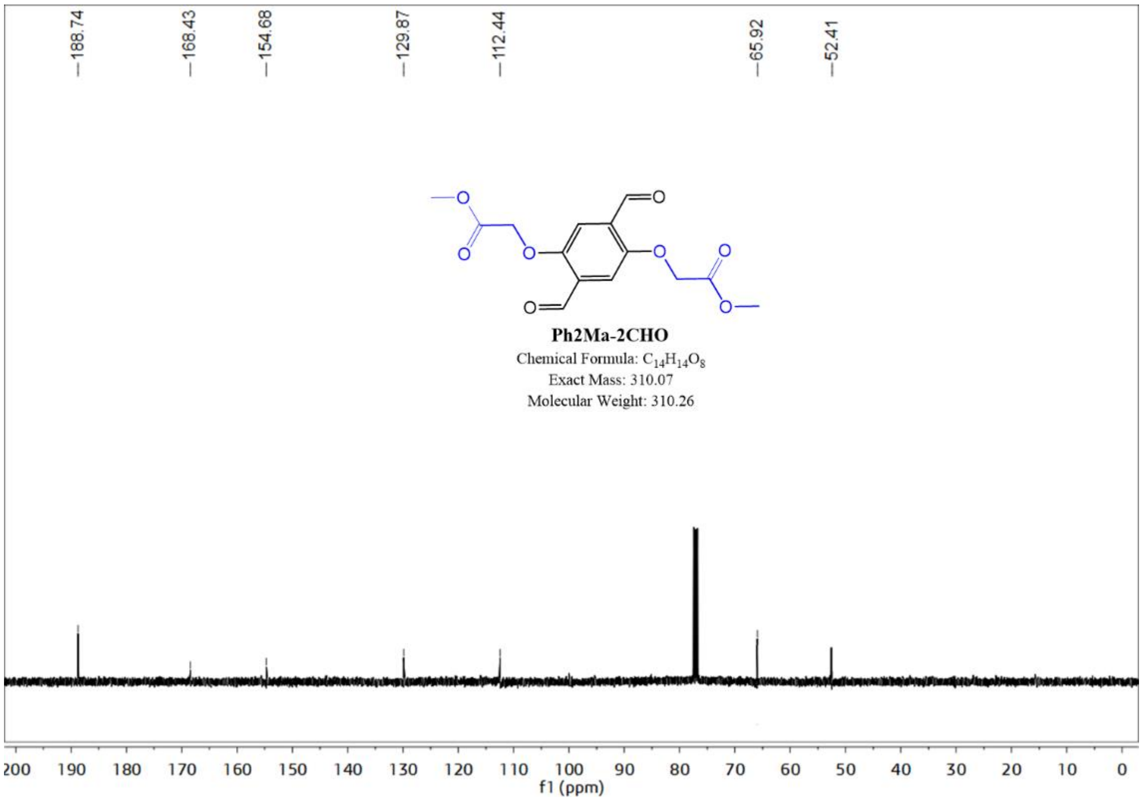

Figure S9. ^{13}C NMR of **Ph2Ma-2CHO**.

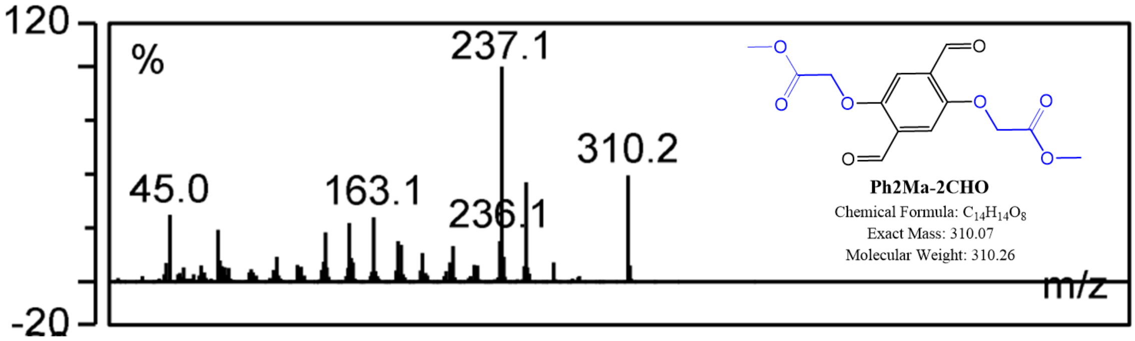

Figure S10. Mass spectrum of **Ph2Ma-2CHO**.

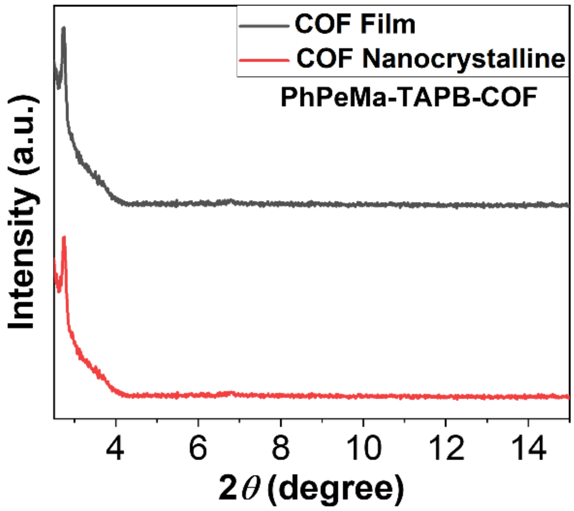

Figure S11. XRD patterns of **PhPeMa-TAPB-COF** film and nanocrystalline powders.

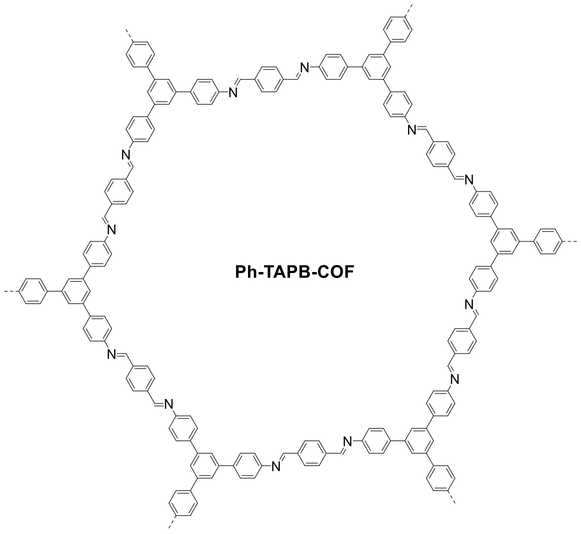

Figure S12. The chemical structure of **Ph-TAPB-COF**.

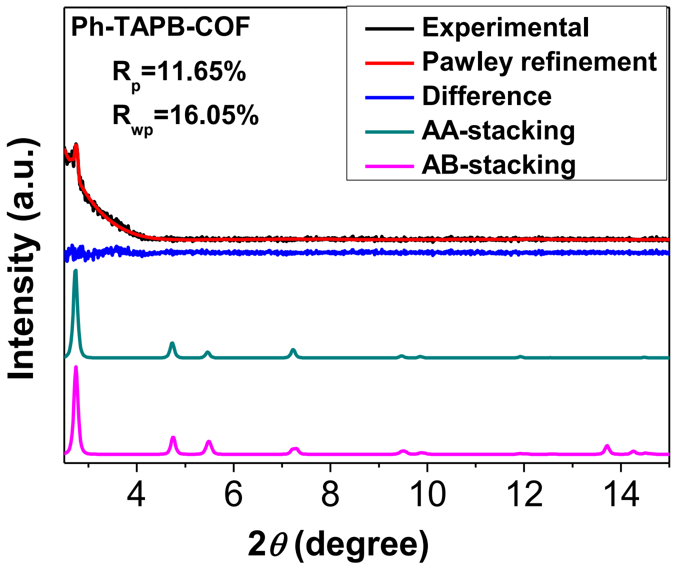

Figure S13. XRD patterns of **Ph-TAPB-COF** film and the simulated patterns.

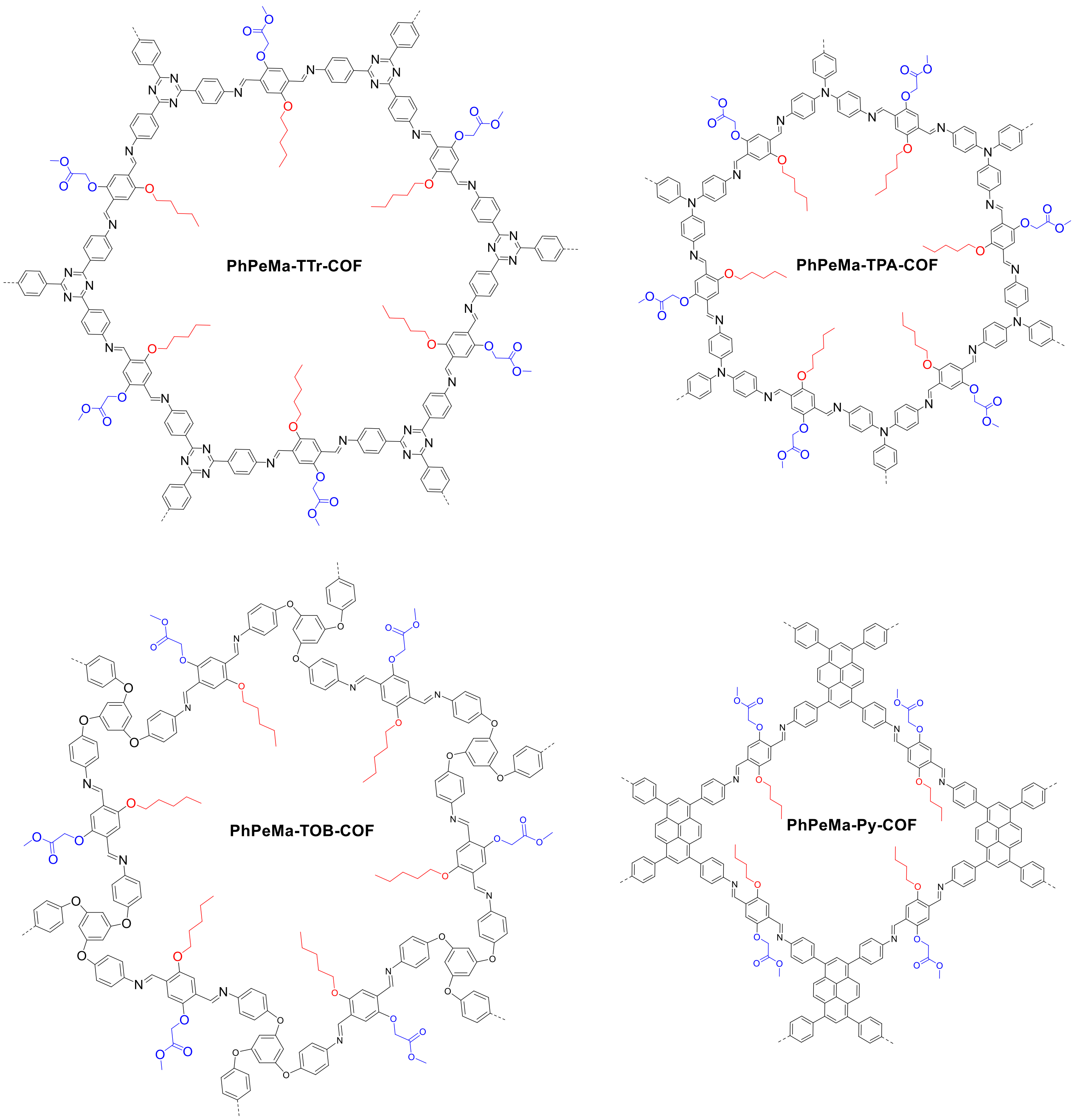

Figure S14. The chemical structure of amphiphilic **PhPeMa-TTr-COF**, **PhPeMa-TPA-COF**, **PhPeMa-TOB-COF**, and **PhPeMa-Py-COF**.

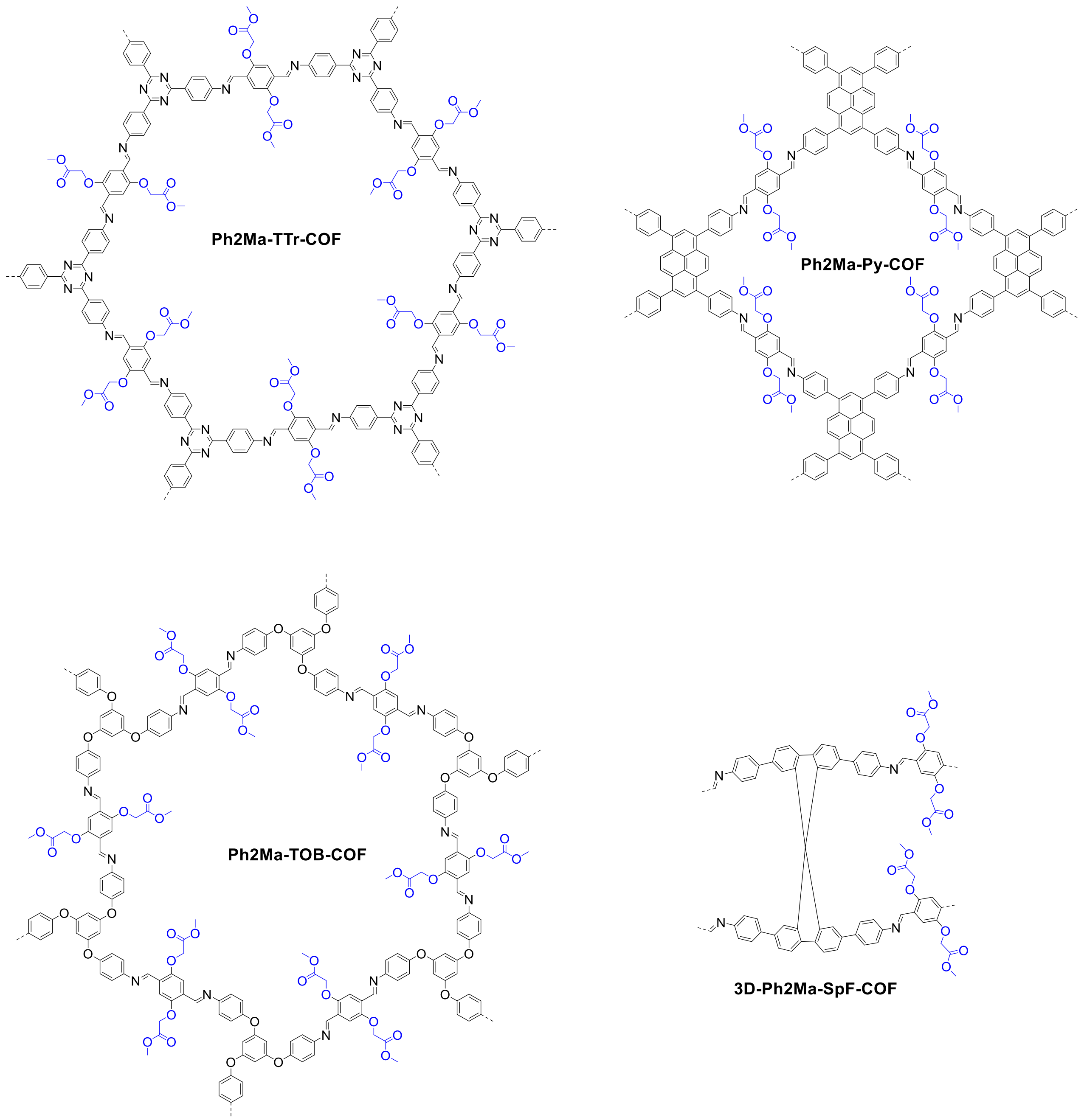

Figure S15. The chemical structure of amphiphilic **Ph2Ma-TTr-COF**, **Ph2Ma-Py-COF**, **Ph2Ma-TOB-COF**, and **3D-Ph2Ma-SpF-COF**.

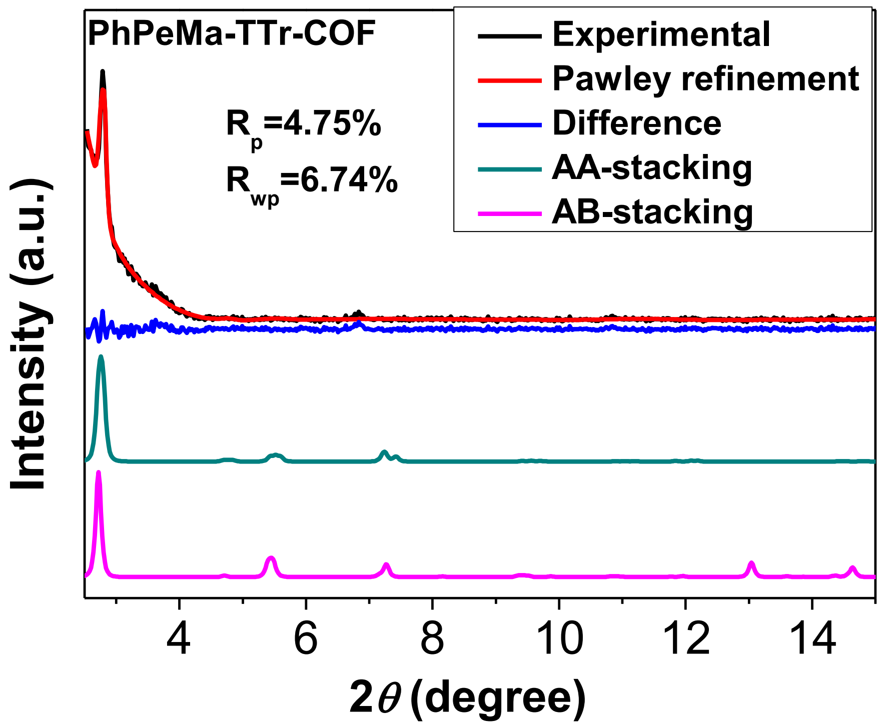

Figure S16. XRD patterns of **PhPeMa-TTr-COF** film and the simulated patterns.

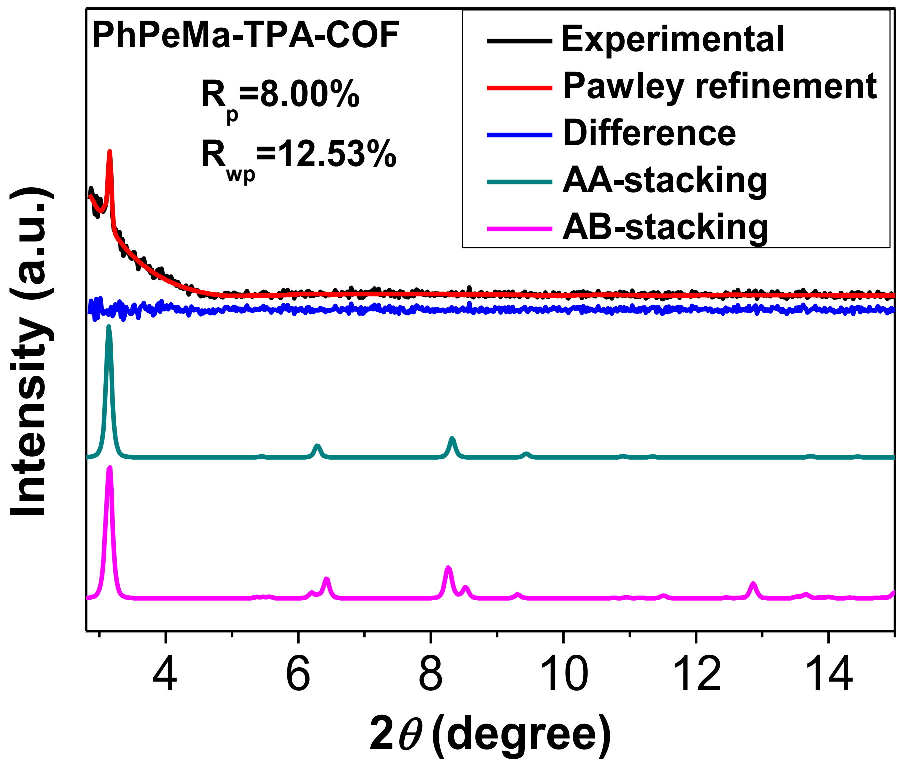

Figure S17. XRD patterns of **PhPeMa-TPA-COF** film and the simulated patterns.

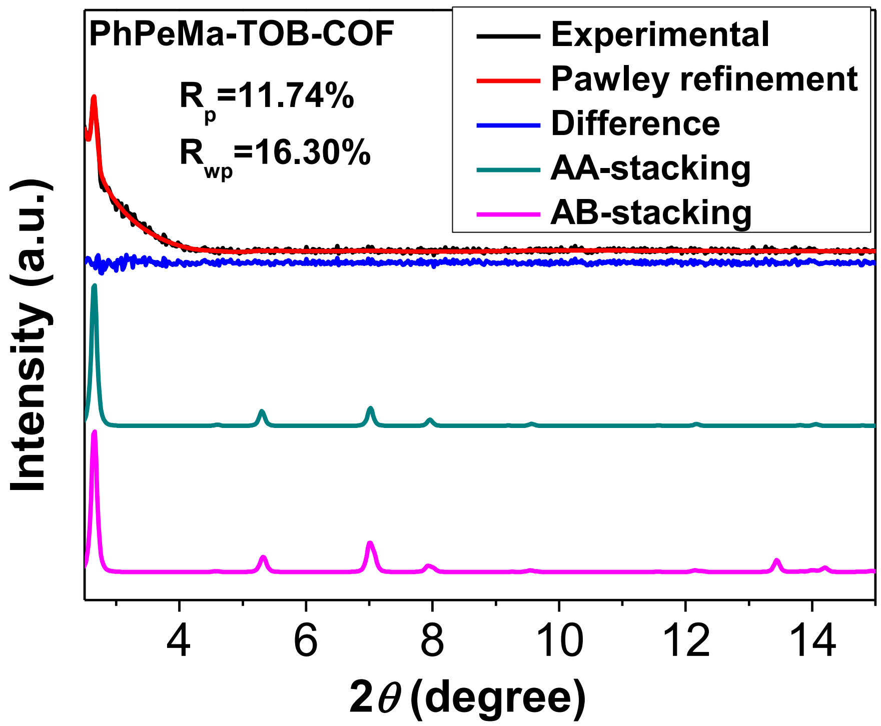

Figure S18. XRD patterns of **PhPeMa-TOB-COF** film and the simulated patterns.

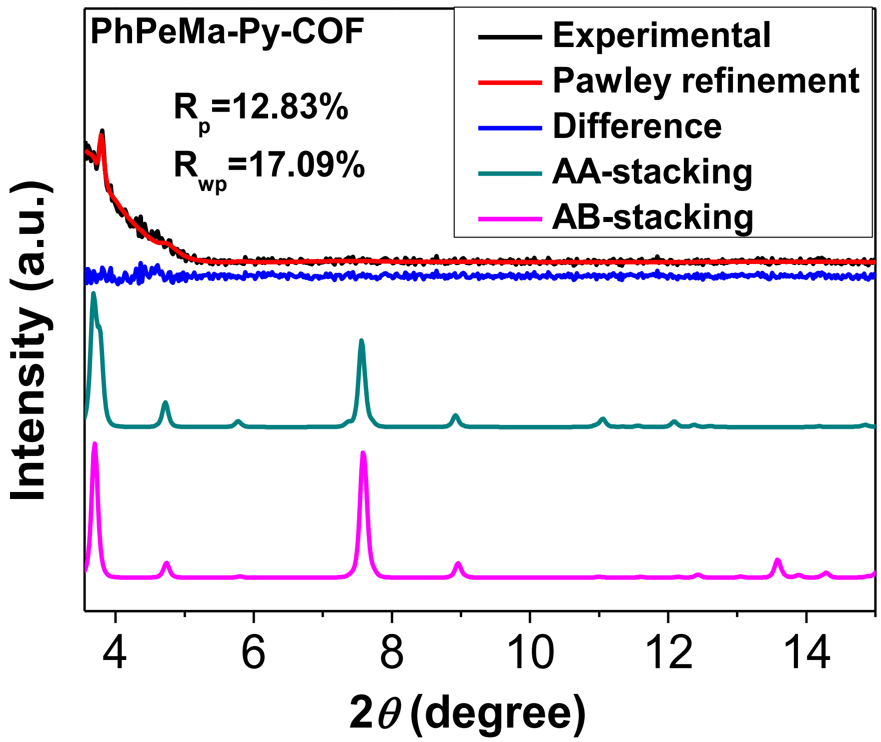

Figure S19. XRD patterns of **PhPeMa-Py-COF** film and the simulated patterns.

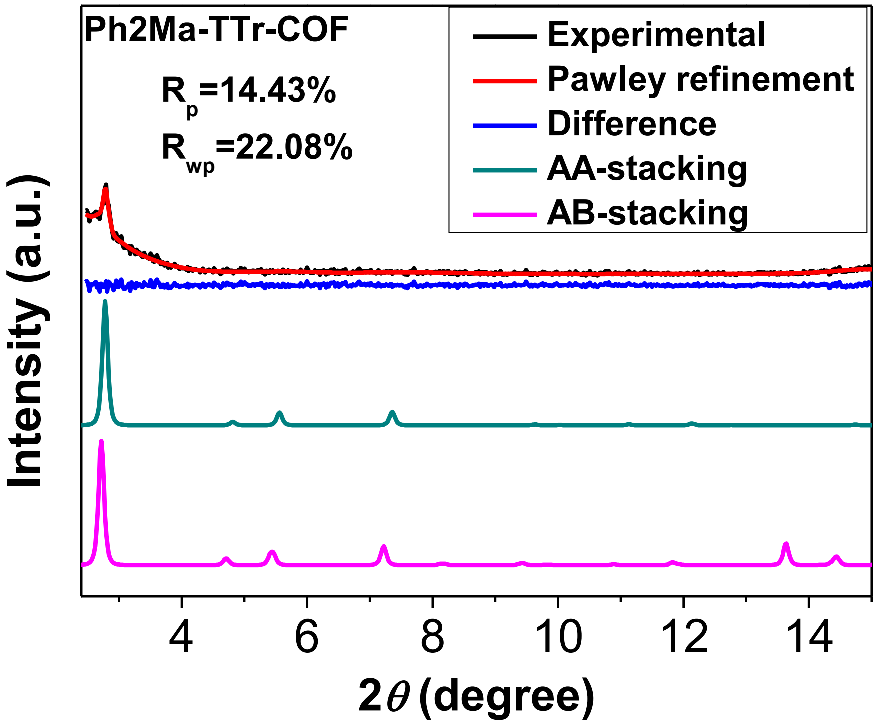

Figure S20. XRD patterns of **Ph2Ma-TTr-COF** film and the simulated patterns.

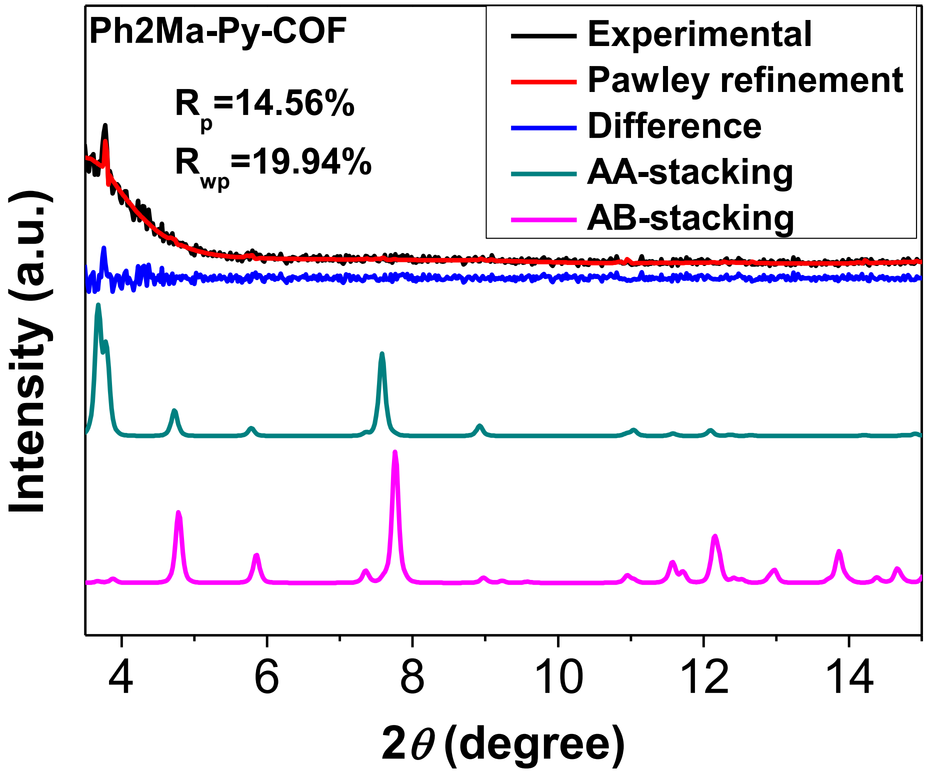

Figure S21. XRD patterns of **Ph2Ma-Py-COF** film and the simulated patterns.

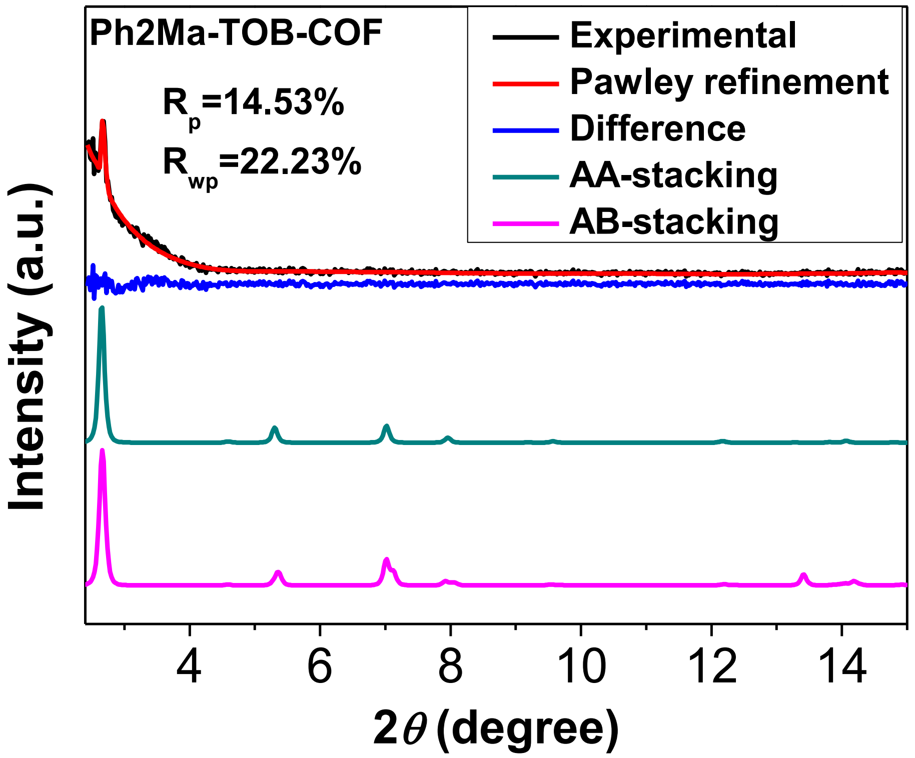

Figure S22. XRD patterns of **Ph2Ma-TOB-COF** film and the simulated patterns.

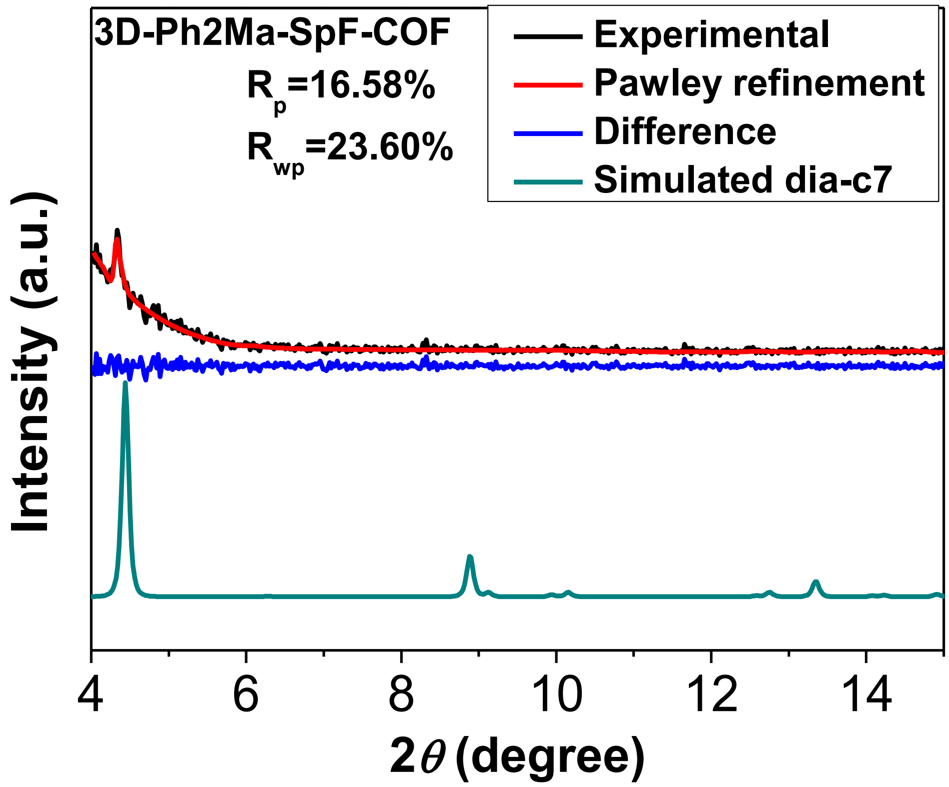

Figure S23. XRD patterns of **3D-Ph2Ma-SpF-COF** film and the simulated patterns.

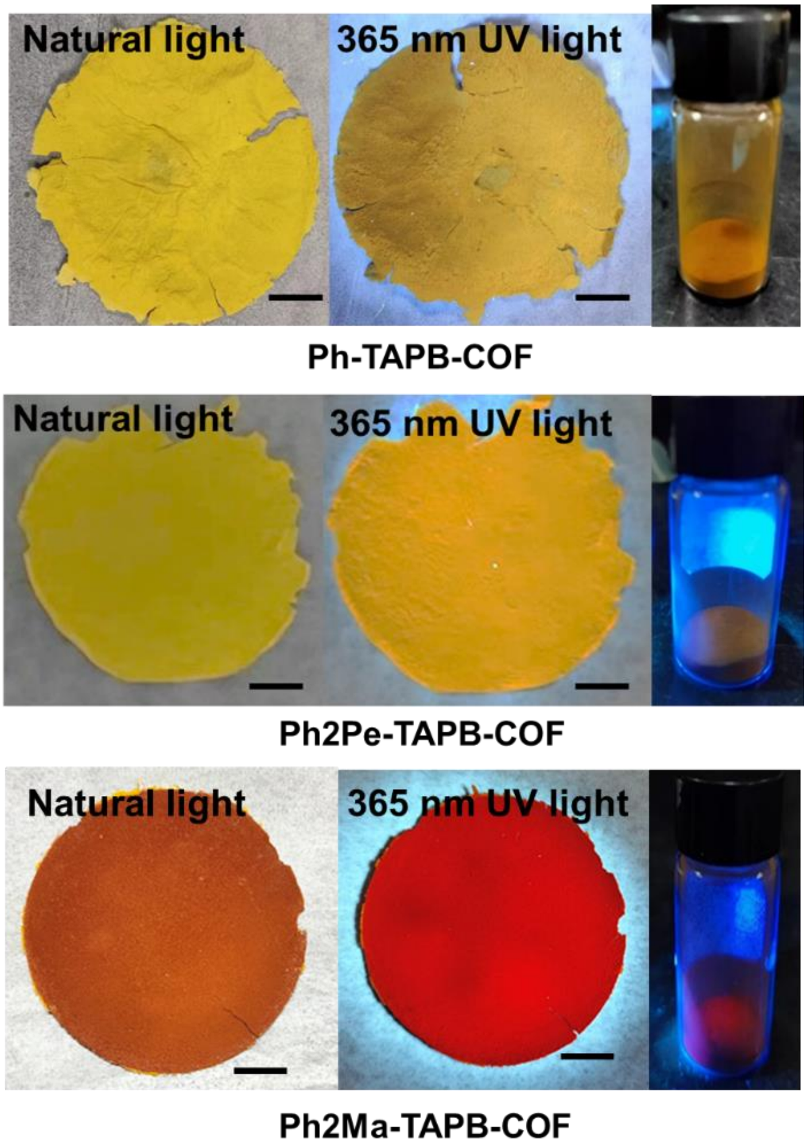

Figure S24. Photographs of **Ph-TAPB-COF**, **Ph2Pe-TAPB-COF**, and **Ph2Ma-TAPB-COF** films under natural light and 365 nm UV light. Bar: 1 cm.

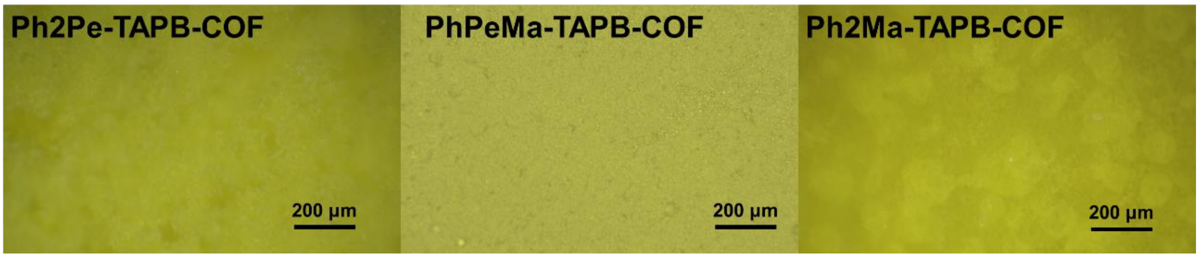

Figure S25. Optical micrographs of **Ph2Pe-TAPB-COF**, **PhPeMa-TAPB-COF**, and **Ph2Ma-TAPB-COF** films.

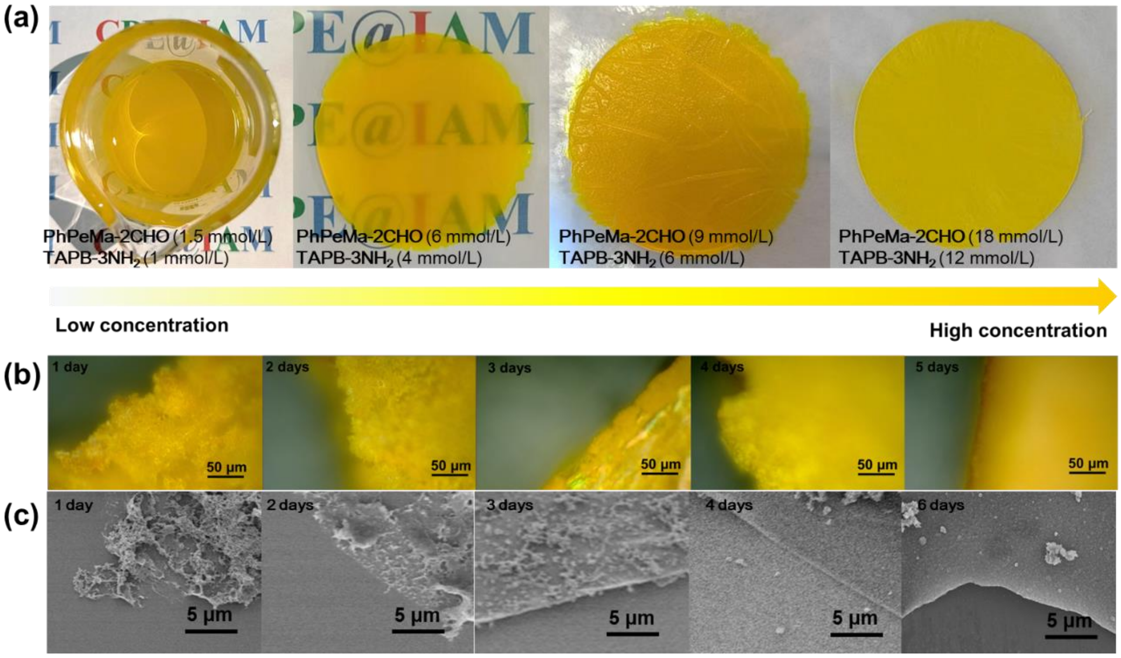

Figure S26. (a) Photographs of **PhPeMa-TAPB-COF** films after five days of reaction with different monomer concentrations. Optical micrographs (b) and SEM images (c) of **PhPeMa-TAPB-COF** films at different reaction times under the same monomer concentration (9 mmol/L **PhPeMa-2CHO** and 6 mmol/L **TAPB-3NH$_2$**).

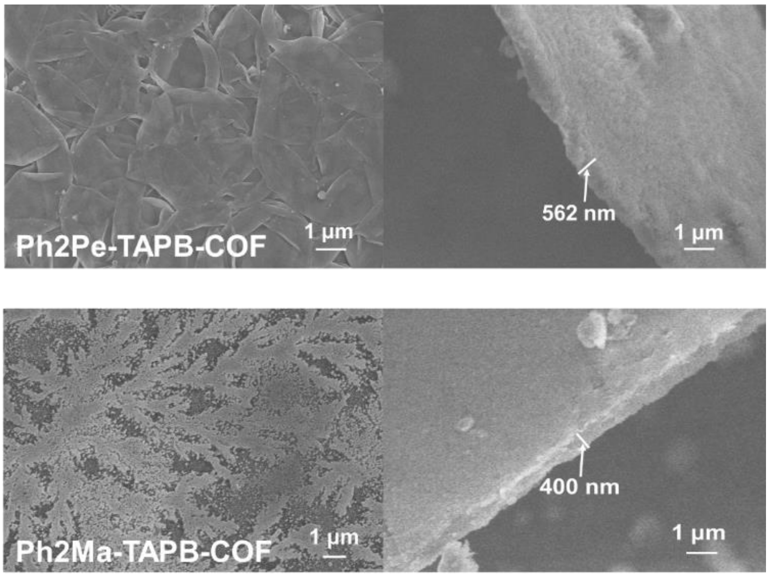

Figure S27. Top-view (left) and cross-sectional view (right) SEM images of **Ph2Pe-TAPB-COF** and **Ph2Ma-TAPB-COF** films.

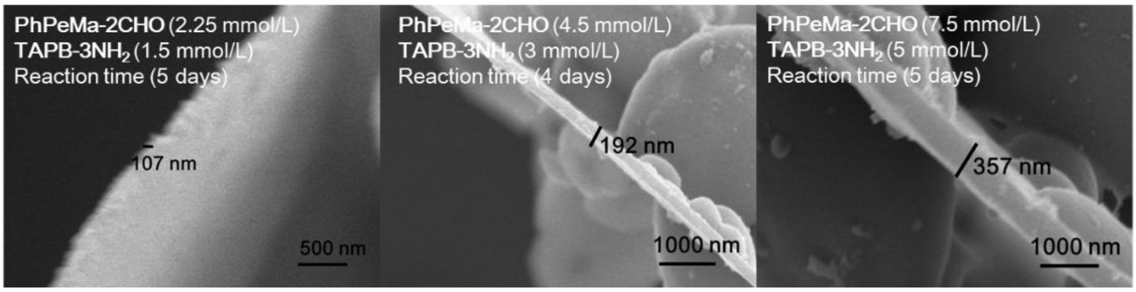

Figure S28. The cross-sectional view SEM images of **PhPeMa-TAPB-COF** films with different monomer concentrations and reaction times.

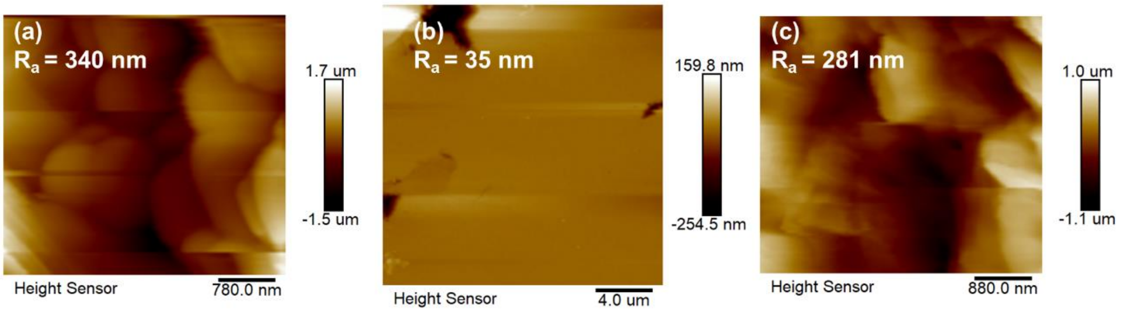

Figure S29. AFM images of **Ph2Pe-TAPB-COF** (a), **PhPeMa-TAPB-COF** (b), and **Ph2Ma-TAPB-COF** films (c).

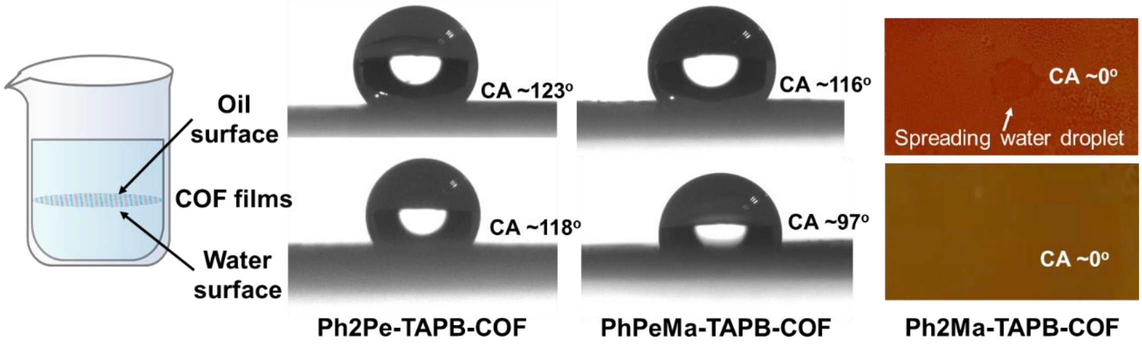

Figure S30. Water contact angles (CA) between the two sides of the **Ph2Pe-TAPB-COF**, **PhPeMa-TAPB-COF**, and **Ph2Ma-TAPB-COF** films.

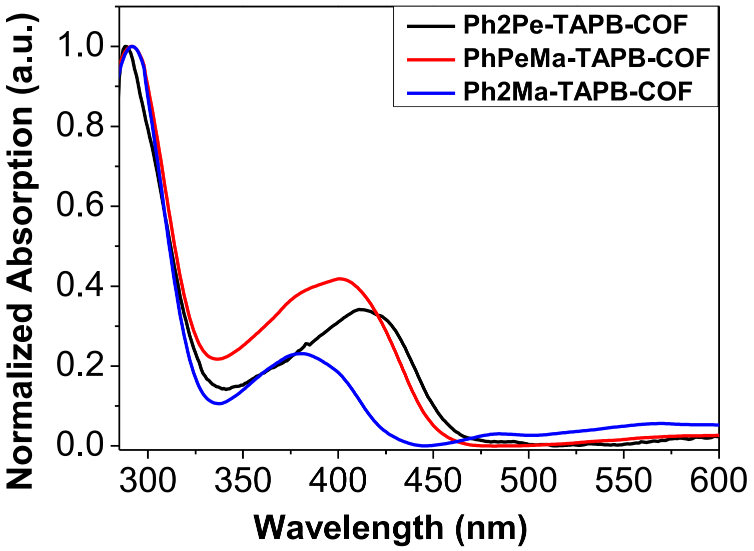

Figure S31. UV-vis spectra of **Ph2Pe-TAPB-COF**, **PhPeMa-TAPB-COF**, and **Ph2Ma-TAPB-COF** films.

Table S1. Photophysical properties of **Ph2Pe-TAPB-COF**, **PhPeMa-TAPB-COF**, and **Ph2Ma-TAPB-COF** films.

	λ_{abs} (nm)	λ_{em} (nm)	PLQY
**Ph2Pe-TAPB-COF**	288, 419	629	0.04
**PhPeMa-TAPB-COF**	289, 404	631	2.38
**Ph2Ma-TAPB-COF**	292, 385	635	1.58

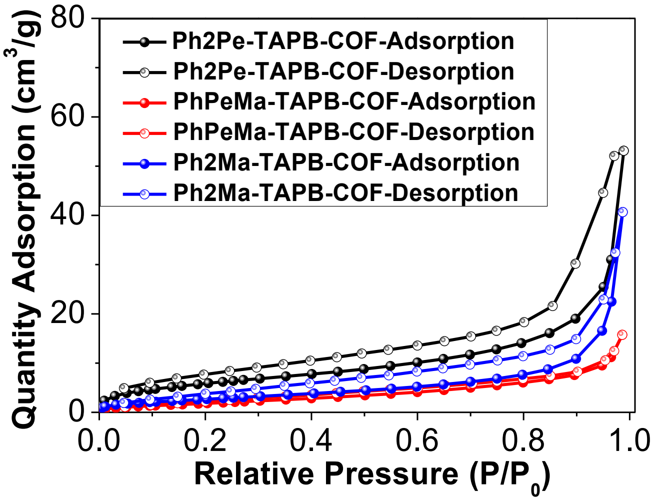

Figure S32. Nitrogen gas adsorption and desorption isotherms of **Ph2Pe-TAPB-COF**, **PhPeMa-TAPB-COF**, and **Ph2Ma-TAPB-COF** films.

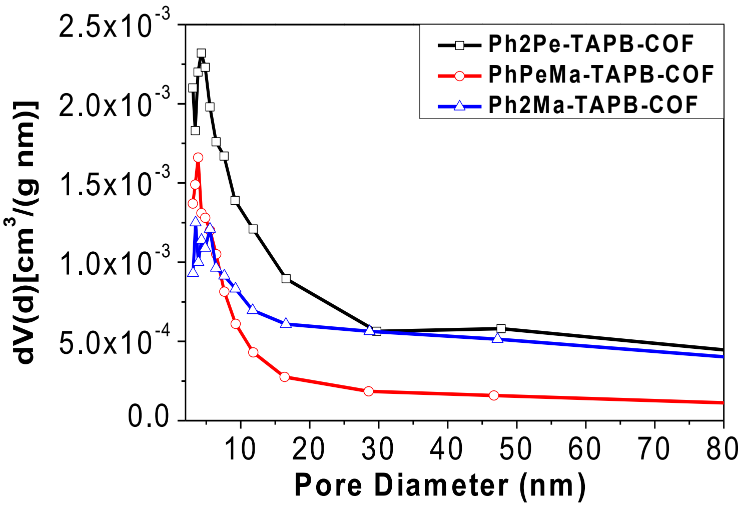

Figure S33. Pore size distribution of **Ph2Pe-TAPB-COF**, **PhPeMa-TAPB-COF**, and **Ph2Ma-TAPB-COF** films.

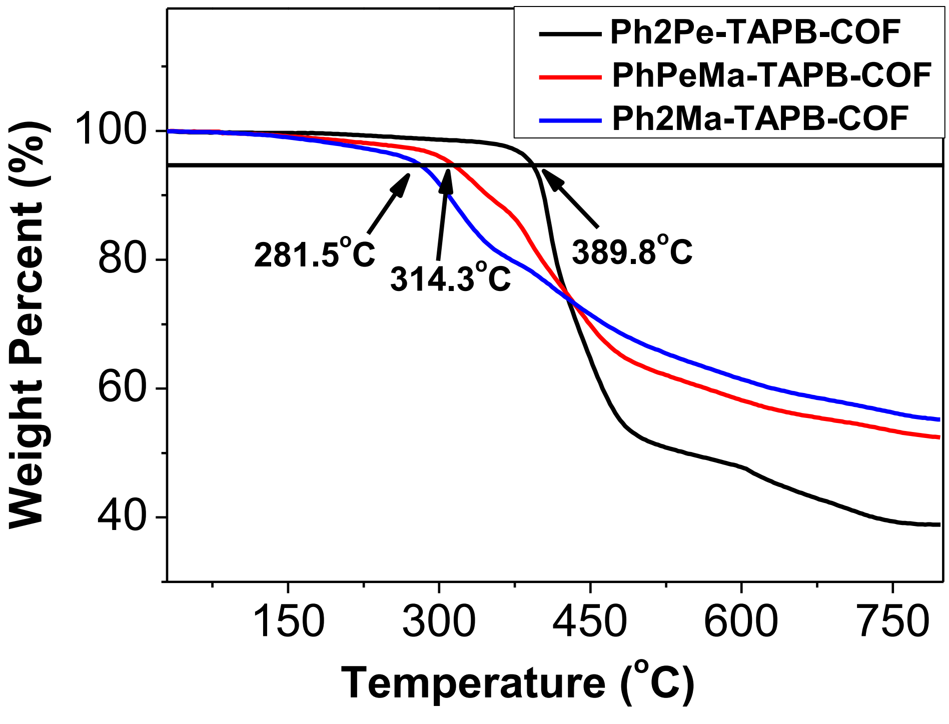

Figure S34. Thermogravimetric analysis (TGA) of **Ph2Pe-TAPB-COF**, **PhPeMa-TAPB-COF**, and **Ph2Ma-TAPB-COF**.

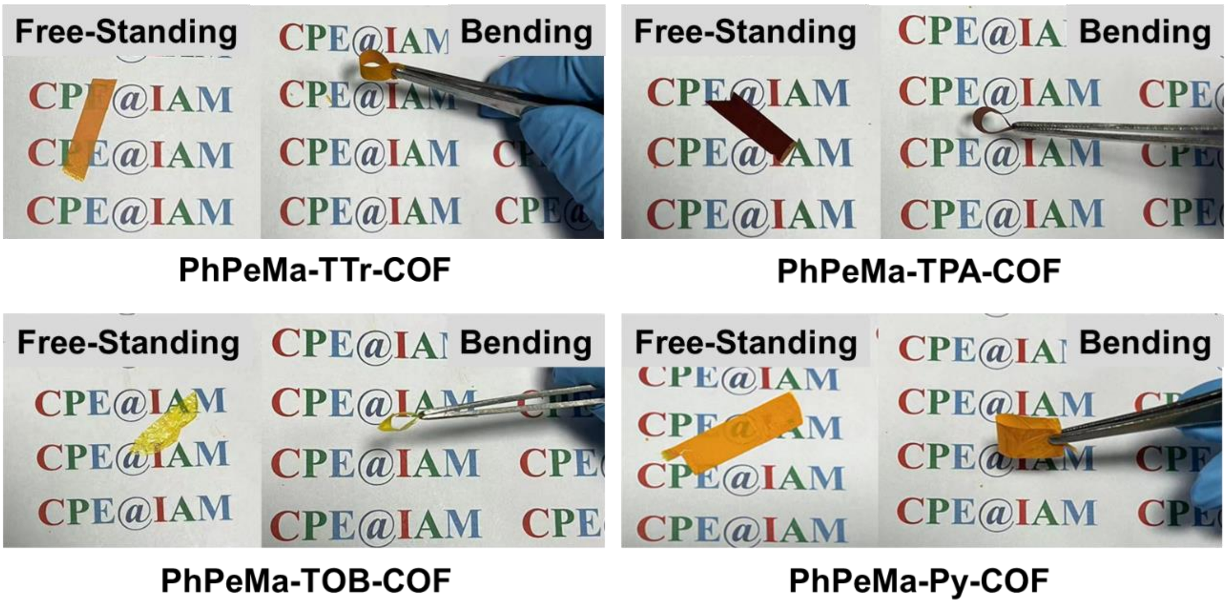

Figure S35. Photographs of the free-standing and bending COF films based on **PhPeMa-TTr-COF**, **PhPeMa-TPA-COF**, **PhPeMa-TOB-COF**, and **PhPeMa-Py-COF**.

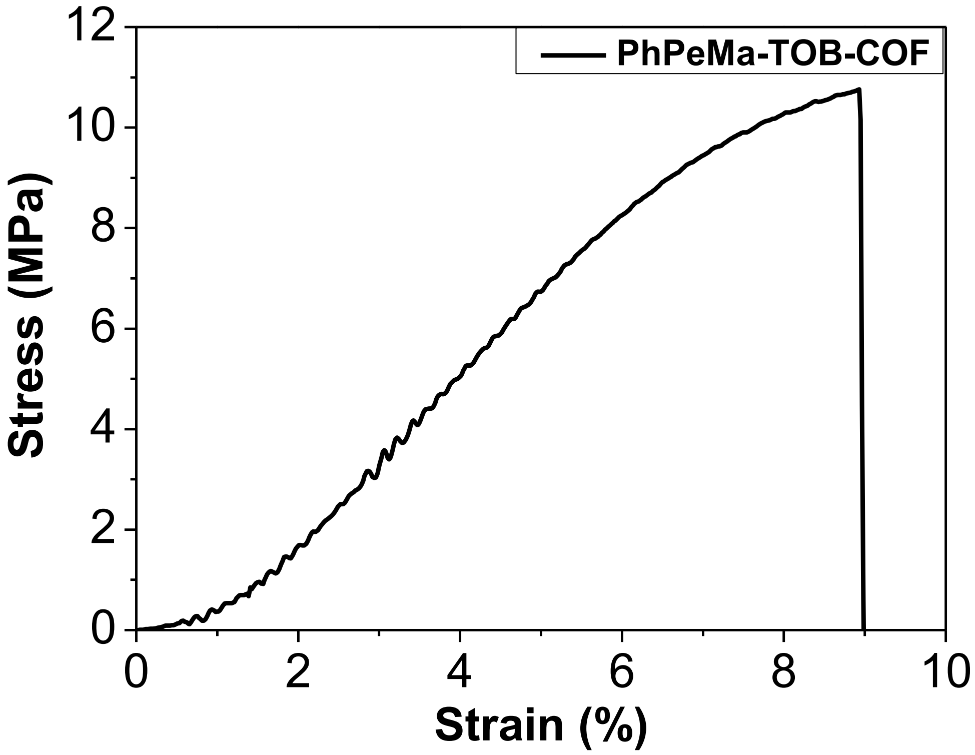

Figure S36. The tensile stress-strain curve of free-standing **PhPeMa-TOB-COF** film.

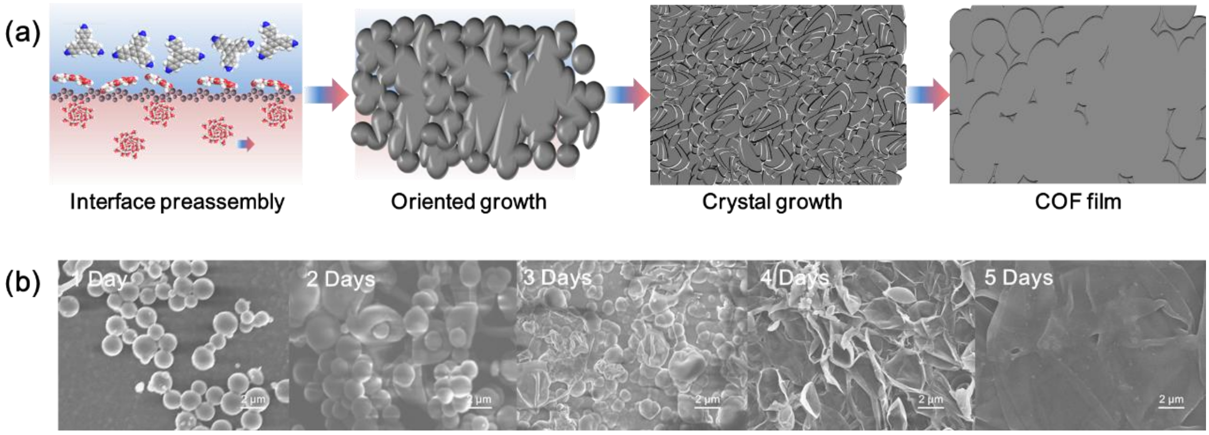

Figure S37. (a) Proposed mechanism for **Ph2Pe-TAPB-COF** film formation using IPOG strategy. (b) Time-dependent SEM study provides support for the proposed mechanism.

Table S2. Performance comparisons of MOF-based and COF-based LED.

Light-Emitting Materials	Device Type	V_{on}[a] (V)	L_{max}[a] (cd/m^2)	CE_{max}[a] (cd/A)	$CD^{[at\ 4\ V]a}$ (mA/cm^2)	CCT (K)	CIE (x, y)	**References**
**PhPeMa-TAPB-COF**	OLED	2.77	1424	0.91	1.92	5584	(0.33, 0.39)[b]	**This Work**
**Ph2Ma-TAPB-COF**	OLED	2.95	1031	0.54	8.80	5266	(0.34, 0.40)[b]	**This Work**
**PhPeMa-Py-COF**	OLED	3.00	532	0.26	9.28	5930	(0.32, 0.39)[b]	**This Work**
**IISERP-COF7**	OLED	--	--	--	~400	--	--	Ref. 1
**Strontium-based MOF**	OLED	--	--	--	~1200	5400	(0.33, 0.33)	Ref. 2
**Zr-NBP MOF**	OLED	3.9	304	0.16	--	--	(0.33, 0.46)	Ref. 3
**3D-TPE-COF**	Photo-luminescence	--	--	--	--	--	(0.30, 0.35)	Ref. 4
**TAT-COF**	Photo-luminescence	--	--	--	--	6500	(0.31, 0.34)	Ref. 5
**viCOF**	Photo-luminescence	--	--	--	--	4463	(0.35, 0.33)	Ref. 6

[a] V_{on}, L_{max}, CE_{max}, and CD stand for turn-on voltage, maximum luminance, maximum current efficiency and current density, respectively. [b] The operating voltages are 14 V, 11 V and 12 V, respectively.

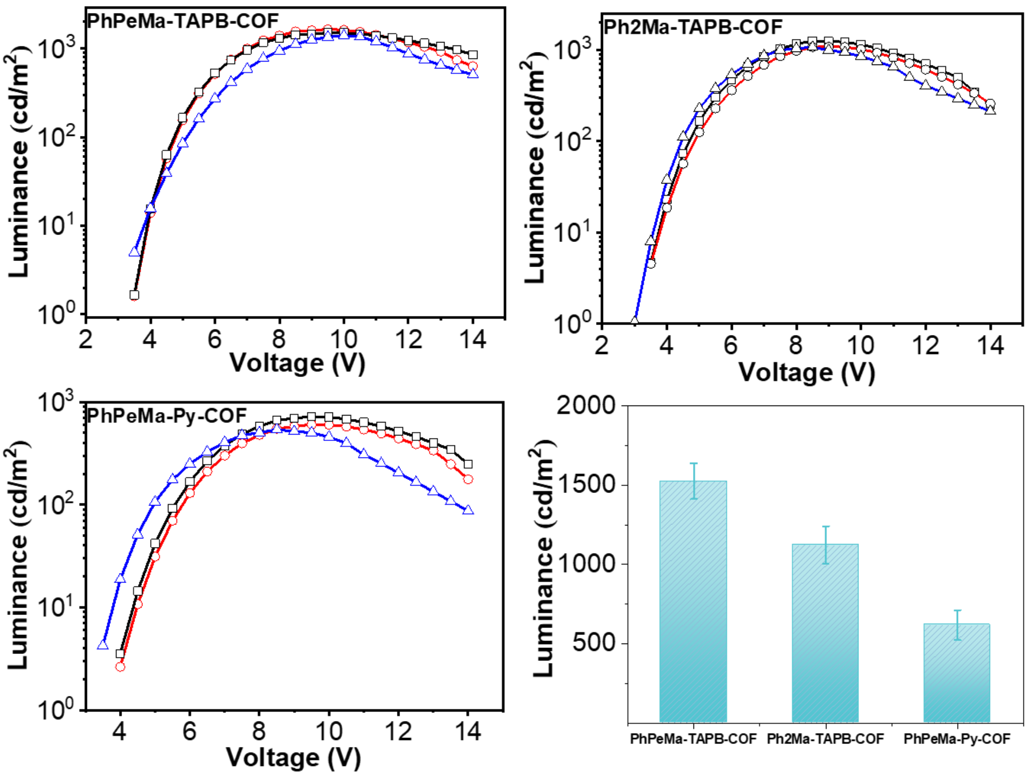

Figure S38. Luminance-voltage (*L-V*) characteristics and error bars of COF-based OLEDs based on different batches of devices.

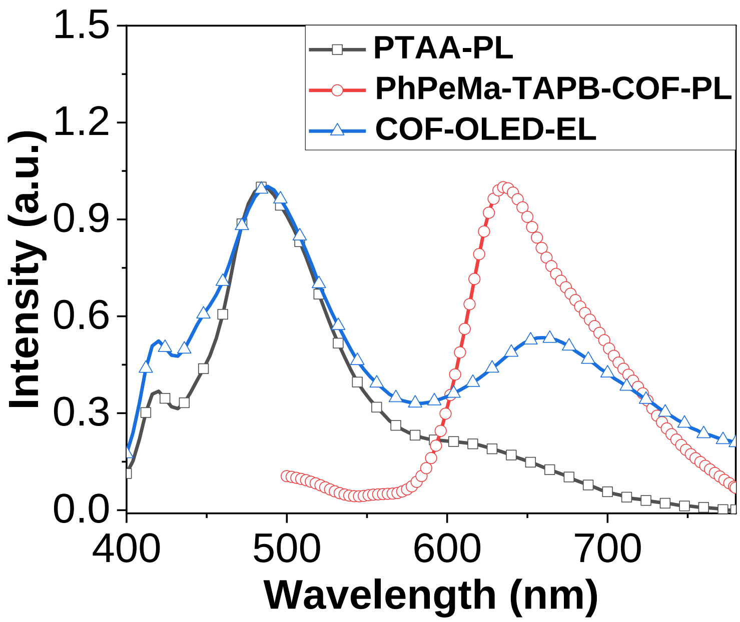

Figure S39. Comparison of the PL spectra of **PTAA** and **PhPeMa-TAPB-COF** with the EL spectra of **PhPeMa-TAPB-COF**-based OLEDs.

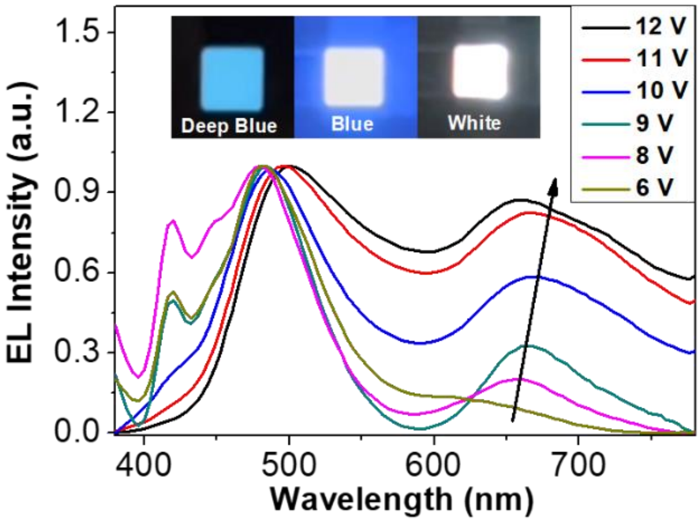

Figure S40. EL spectra of **Ph2Ma-TAPB-COF**-based OLEDs with different driving voltages.

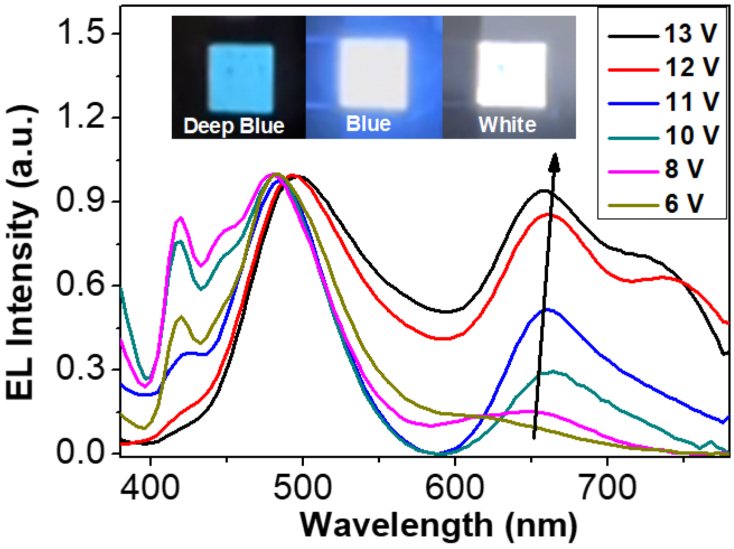

Figure S41. EL spectra of **PhPeMa-Py-COF**-based OLEDs with different driving voltages.

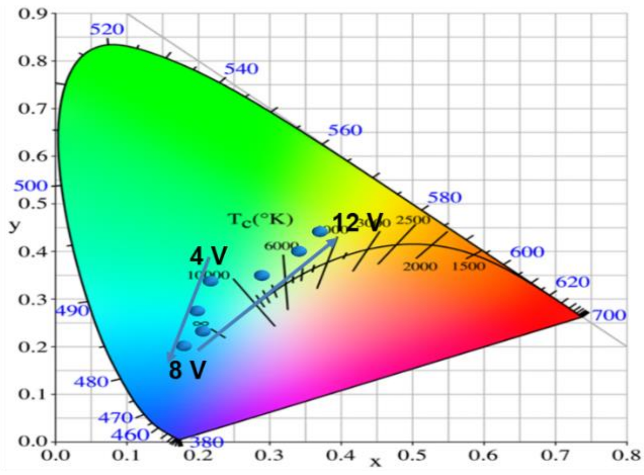

Figure S42. CIE diagram of **Ph2Ma-TAPB-COF**-based OLEDs with different driving voltages.

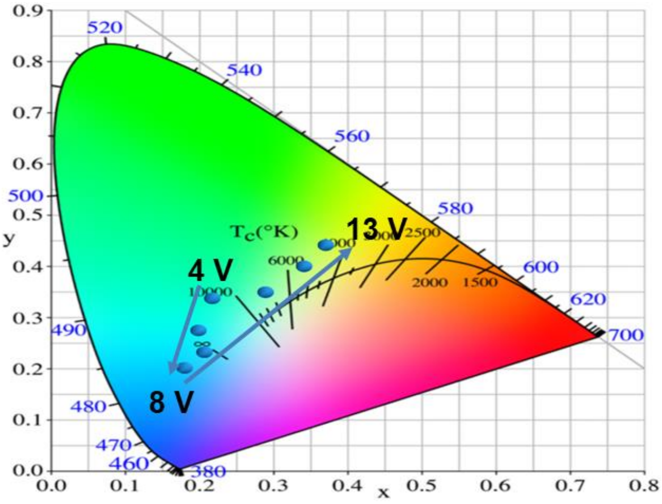

Figure S43. CIE diagram of **PhPeMa-Py-COF**-based OLEDs with different driving voltages.

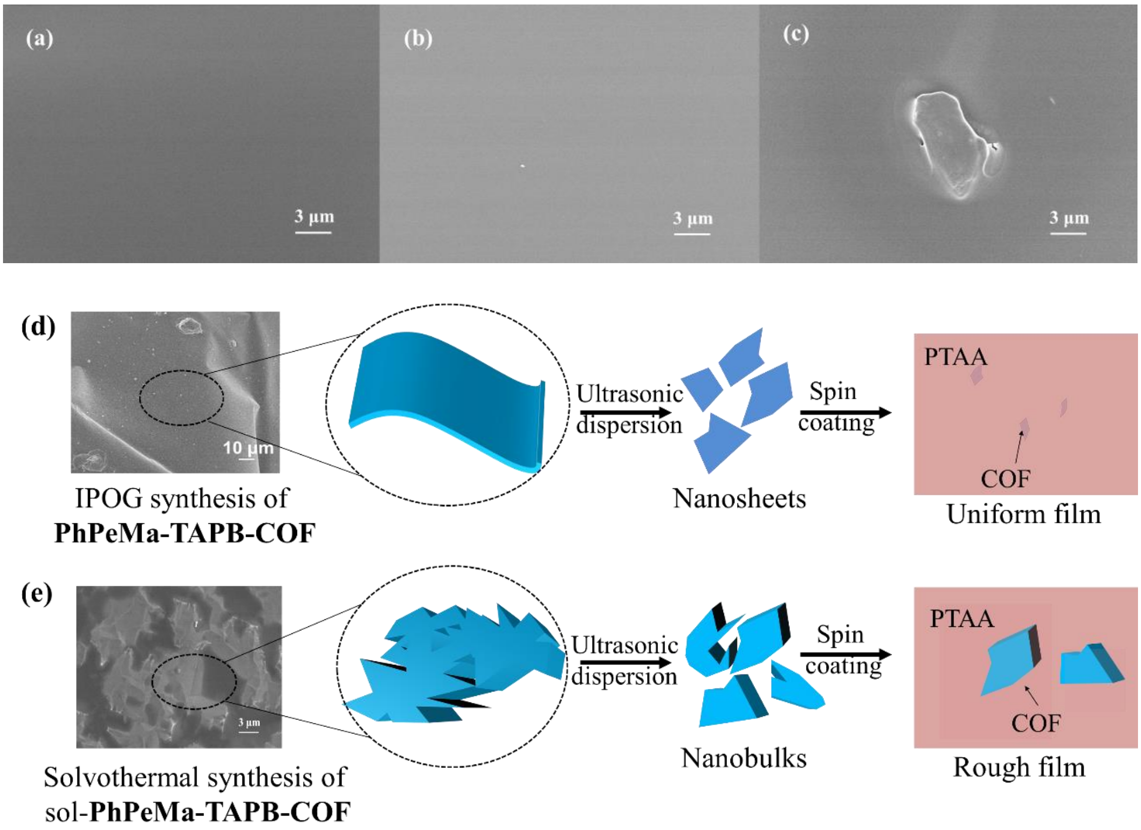

Figure S44. SEM images of **PTAA** films (a), **PTAA/PhPeMa-TAPB-COF** (IPOG synthesis) films (b), **PTAA**/sol-**PhPeMa-TAPB-COF** (Solvothermal synthesis) films (c). Film spin-coating process of **PhPeMa-TAPB-COF** (d) and sol-**PhPeMa-TAPB-COF** (e). The left picture is SEM images of **PhPeMa-TAPB-COF** films and sol-**PhPeMa-TAPB-COF** powders.

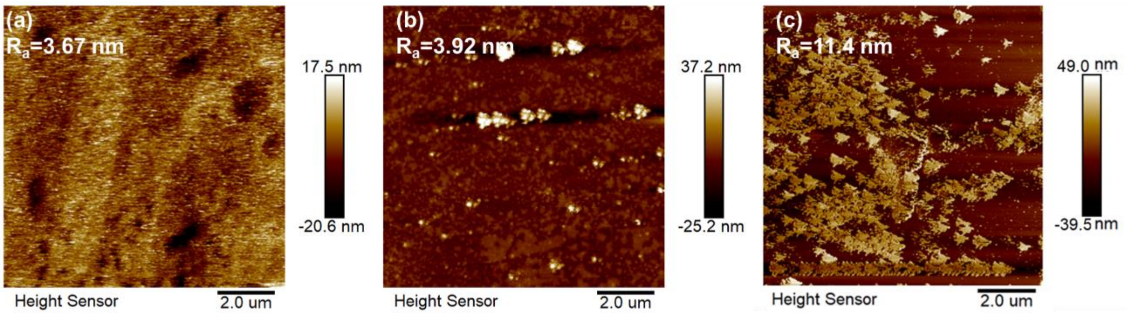

Figure S45. AFM images of **PTAA** films (a), **PTAA/PhPeMa-TAPB-COF** (IPOG synthesis) films (b), and **PTAA**/sol-**PhPeMa-TAPB-COF** (Solvothermal synthesis) films (c).

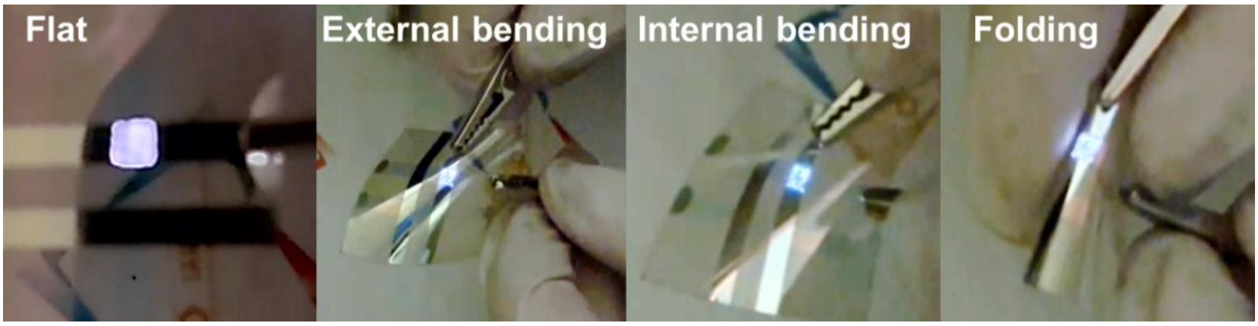

Figure S46. Photographs of flexible OLEDs based on **PhPeMa-TAPB-COF** under the condition of flat, external bending, internal bending and folding in half.

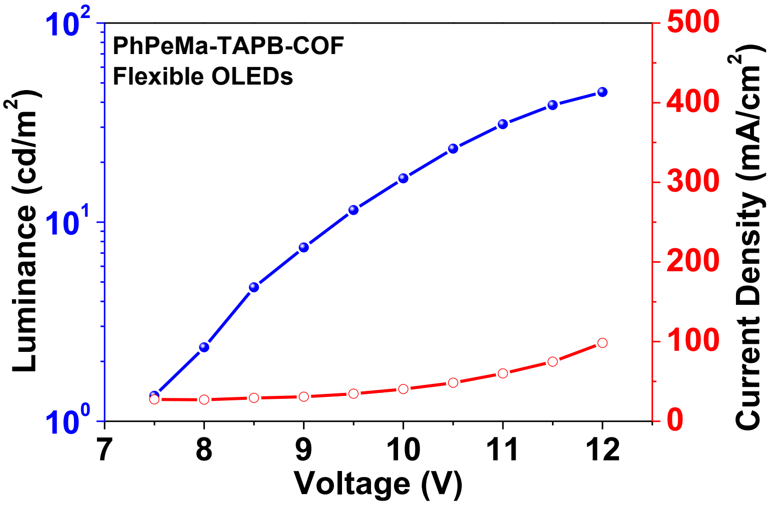

Figure S47. Current density-luminance-voltage (*J-L-V*) characteristics of COF-based flexible OLEDs.

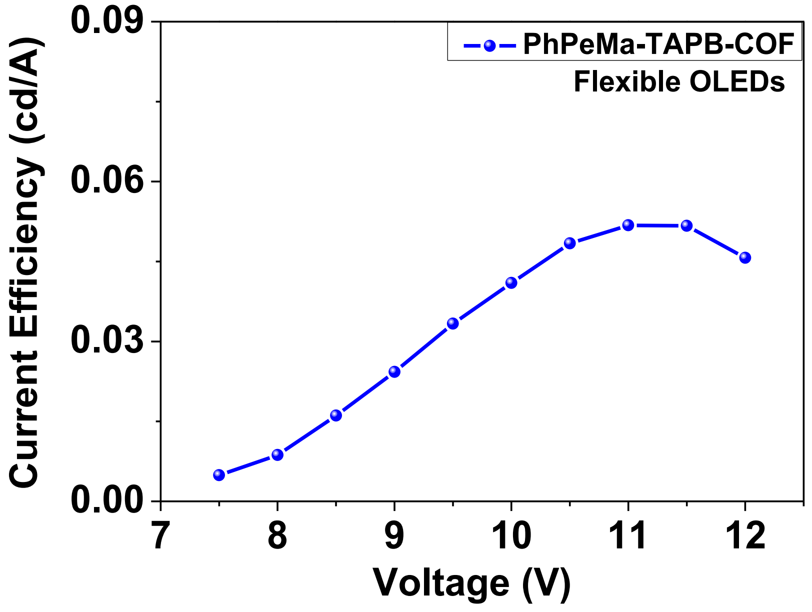

Figure S48. Current efficiency-voltage curves of COF-based flexible OLEDs.

References

[1] Haldar, S.; Chakraborty, D.; Roy, B.; Banappanavar, G.; Rinku, K.; Mullangi, D.; Hazra, P.; Kabra, D.; Vaidhyanathan, R., Anthracene-resorcinol derived covalent organic framework as flexible white light emitter. *J. Am. Chem. Soc.,* **2018,** *140*, 13367-13374.

[2] Haider, G.; Usman, M.; Chen, T. P.; Perumal, P.; Lu, K. L.; Chen, Y. F., Electrically driven white light emission from intrinsic metal-organic framework. *ACS Nano,* **2016,** *10*, 8366-8375.

[3] Kaiyasuan, C.; Somjit, V.; Boekfa, B.; Packwood, D.; Chasing, P.; Sudyoadsuk, T.; Kongpatpanich, K.; Promarak, V., Intrinsic hole mobility in luminescent metal-organic frameworks and its application in organic light-emitting diodes. *Angew. Chem. Int. Ed.,* **2022,** *61*, e202117608.

[4] Ding, H.; Li, J.; Xie, G.; Lin, G.; Chen, R.; Peng, Z.; Yang, C.; Wang, B.; Sun, J.; Wang, C., An AIEgen-based 3D covalent organic framework for white light-emitting diodes. *Nat. Commun.,* **2018,** *9*, 5234.

[5] Yang, S. Z.; Streater, D.; Fiankor, C.; Zhang, J.; Huang, J. E., Conjugation- and aggregation-directed design of covalent organic frameworks as white-light-emitting diodes. *J. Am. Chem. Soc.,* **2021,** *143*, 1061-1068.

[6] Wang, Y.; Cheng, Y. Z.; Wu, K. M.; Yang, D. H.; Liu, X. F.; Ding, X. S.; Han, B. H., Linkages make a difference in the photoluminescence of covalent organic frameworks. *Angew. Chem. Int. Ed.,* **2023,** *62*, e202310794.
